# Liver Fibrosis: Molecular Pathogenesis and Therapeutic Interventions

**DOI:** 10.1002/mco2.70750

**Published:** 2026-05-03

**Authors:** Jiaorong Qu, Wenqing Qin, Minghang Dong, Zhi Ma, Si Li, Runping Liu, Ranyun Chen, Changmeng Li, Xiaojiaoyang Li

**Affiliations:** ^1^ Beijing University of Chinese Medicine Beijing China

**Keywords:** drug therapy, etiological treatment, future therapeutic directions, liver fibrosis, pathogenic mechanisms

## Abstract

Liver fibrosis is a common pathological process, leading to the development of end‐stage liver diseases. It is triggered by various etiological drivers including viral hepatitis, metabolic‐associated steatotic liver disease (MASLD), and cholestasis. Given the substantial impact of liver fibrosis on individuals and its associated mortality rates, effective management of this condition is crucial for improving public health. Despite a growing number of preclinical studies and clinical trials, a systematic synthesis remains lacking. In this review, the molecular panorama of liver fibrogenesis is summarized at first, encompassing etiological drivers of chronic liver injury, key cellular players, core signaling pathways, and extracellular matrix dynamics. Therapeutic interventions in preclinical or clinical stages are systematically classified into two main categories: etiological treatment as the foundational approach and mechanism‐based antifibrotic therapies. Emerging and future therapeutic strategies, including those targeting gut–liver axis, gut microbiota, and cell‐based therapies, are also addressed along with inherent challenges. Furthermore, future perspectives centered on precision medicine, combination therapies, novel target discovery, and advanced drug delivery systems are emphasized. This review offers a comprehensive overview of the etiologies, diagnostic approaches, pathogenic mechanisms, current development of antifibrotic agents, and prospects for future therapeutic directions of liver fibrosis.

## Graphical abstract

We systematically summarized the etiologies, diagnostic approaches, and pathogenic mechanisms of liver fibrosis. Also, the therapeutic interventions for liver fibrosis were systematically classified into two main categories: etiological treatment and mechanism‐based antifibrotic therapies.

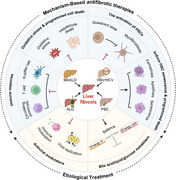

## 1 Introduction

Chronic liver disease is a pressing global public health concern, with the number of affected individuals increasing by 13% since 2000 [1]. Almost all cases of chronic liver disease progression accompanied by liver fibrosis, a pathological repair process triggered by persistent liver injury [2, 3]. The pathophysiological mechanisms of liver fibrosis primarily involve inflammatory reactions, collagen synthesis and secretion, extracellular matrix (ECM) remodeling, fibrous tissue proliferation, and nodular regeneration of hepatocytes. If the liver fibrotic process persists over time, chronic liver diseases will progress to irreversible cirrhosis and even hepatocellular carcinoma (HCC) [4, 5]. Emerging trial data now affirm that liver fibrosis, once deemed irreversible, can regress with targeted therapy [6, 7].

While lifestyle changes are generally considered safe and effective in managing liver fibrosis, patients, especially those with concurrent underlying metabolic disorders, may struggle to maintain long‐term adherence or achieve desired results within the expected timeframe, particularly in the advanced stages of the disease. In this context, rational pharmacological interventions are indispensable and are expected to influence all key steps in liver fibrosis progression. But at present, only resmetirom has been approved by United States Food and Drug Administration (US FDA) for the treatment of metabolic dysfunction‐associated steatohepatitis (MASH)‐associated liver fibrosis. This suggested that further work should focus on identifying novel therapeutic targets of fibrosis, which is essential in mitigating the impact of chronic liver disease on morbidity and mortality [8]. Thus, it is crucial to systematically summarize the molecular pathogenesis and therapeutic interventions about live fibrosis.

In this review, we first clarify the etiological drivers of liver fibrosis and the specifical mechanism by which these drivers initiate and promote liver fibrosis. Then, the current status and latest research progress of diagnostic and assessment strategies are introduced. Moreover, we provide a systematic summary of current therapeutic strategies and their corresponding drug development stages. We then categorized candidate drugs into “etiological treatment” and “targeted antifibrotic therapies (mechanism‐based)”. Notably, the therapeutic strategies target different drivers of liver fibrosis exhibit disease heterogeneity and are summarized in section *Etiological Treatment*. In section *Targeted Antifibrotic Therapies (Mechanism‐Based)*, regardless of the etiology, the therapeutic strategies targeting the common mechanisms of liver fibrosis are summarized. Subsequently, challenges in the development of antifibrotic drug and future perspectives were discussed.

## 2 Molecular Pathogenesis of Liver Fibrosis

To better understand the occurrence and development of liver fibrosis, the etiological drivers including metabolic‐associated steatotic liver disease (MASLD), viral hepatitis, and cholestasis that trigger liver fibrosis were discussed. Furthermore, initiated by factors descripted above, we further discuss how key cell types such as hepatocytes, cholangiocytes, immune cells, and hepatic stellate cells (HSCs) promote the progression of liver fibrosis, as well as the related core pathways and molecular mechanisms. Moreover, as one of the basic characteristics of liver fibrosis, the changes and modulation of ECM dynamics play key roles in the progression of liver fibrosis and can serve as therapeutic targets.

### 2.1 Etiological Drivers of Chronic Liver Injury

Metabolic dysfunction‐associated steatotic liver disease (MASLD) is a prevalent chronic liver condition. Obesity and adiposopathy are the primary causes of MASLD, along with associated metabolic disturbances such as insulin resistance, Type 2 diabetes mellitus (T2DM), dyslipidemia, and hypertension [9]. The dysfunction of lipid metabolism is a represented characteristic in MASLD. The accumulation of fat in the liver and the development of MASLD involve increased fatty acid (FA) production from dietary sources, de novo lipogenesis, and lipolysis in adipose tissue. Additionally, the reasons that contribute to lipid dysregulation inlcude enhanced FA uptake, abnormalities in FA synthase and FA β‐oxidation, as well as elevated hepatic cholesterol concentrations [10]. Lipid overload‐induced lipotoxicity further induces oxidative stress, inflammation, hepatocyte death, HSC activation, and other pathological changes, which promote the progression of MASLD to MASH and liver fibrosis [11].

The development of ALD is influenced by multiple factors. The most important one is the direct toxic effects of ethanol and its primary metabolite, acetaldehyde, causing oxidative stress, inflammation, and direct hepatocyte damage [12]. Apart from the toxic effects of alcohol metabolites, significant changes in the gut microbiome and the release of endotoxins also play a crucial role in inducing inflammatory response in ALD. These mechanisms interact to amplify liver damage by activating Kupffer cells (KCs) and recruiting neutrophils.

Viral hepatitis results from infection by various hepatitis viruses, each of which has unique structural characteristics and replication strategies. For example, hepatitis B viruses (HBV) and hepatitis D viruses enter liver cells via the sodium taurocholate cotransporting polypeptide receptor, which facilitates internalization through endocytosis [13]. After entry into the cell, these viruses move to the nucleus, where their double‐stranded relaxed circular DNA is converted into covalently closed circular DNA. Then, with the assistance of DNA polymerase, new viruses are continuously replicated. The innate immune system, as the first line of defense, can rapidly detect the amplified virus [14]. Liver damage caused by viral hepatitis is primarily mediated by the host immune response, rather than direct cytopathic effects of the virus. The immune system causes damage to liver tissue when it attempts to eliminate liver cells that infected with viruses. Specifically, cytotoxic T lymphocytes (CTLs) recognize viral antigens on infected hepatocytes, leading to apoptosis of these cells [15]. However, this immune response also induces inflammation and necrosis that are the main reason lead to liver fibrosis and cirrhosis. In addition to CTL‐mediated damage, the inflammatory response induced by immune cell‐released cytokines such as interferons, tumor necrosis factor‐α (TNF‐α), and interleukins can promote hepatocyte apoptosis and activate HSCs to aggravate liver injury and enhance ECM production [16].

Cholestasis occurs when bile flow through the intrahepatic or extrahepatic bile ducts is reduced. This usually results from impaired bile acid synthesis, secretion, and excretion. Persistent cholestasis causes cholestatic liver injury, which mainly includes two major subtypes: primary biliary cirrhosis (PBC) and primary sclerosing cholangitis (PSC). The etiology of cholestatic liver injury involves several factors, such as genetic factors, cholelithiasis, schistosomiasis, progressive destruction of the bile duct, and persistent intrahepatic inflammation [17]. The bile acids accumulation in the liver can induce oxidative stress damage in hepatocytes and promote the release of damage‐associated molecular patterns (DAMPs). Toll‐like receptors can response to DAMPs, promoting the expression of key inflammatory factors and activating related inflammatory signaling pathways in hepatocytes [18]. Furthermore, hepatocyte specific inflammatory responses promote the infiltration of bone marrow‐derived macrophages into the nonparenchymal regions of the liver, resulting in the disruption of bile flow and bile acid metabolism. In addition, elevated bile acid levels in the liver induce ductular reaction, characterized by cholangiocyte proliferation, inflammatory cell infiltration, and periportal fibrosis [19]. The ductular reaction can further induce the secretion of cytokines such as transforming growth factor‐β (TGF‐β) by cholangiocytes, which in turn activates HSCs.

Fibrosis in MASLD, viral hepatitis, and cholestatic diseases have different drivers. Although, metabolic dysfunction, inflammation, and cholestasis are all involved in fibrosis induced by different etiologies, their contributions to disease progression vary by etiology. For instance, targeting lipid/glucose metabolism may be a more effective strategy for alleviating liver fibrosis in patients with MASLD and MASH. In patients with PBC and PSC, regulating bile acid metabolism could prevent liver fibrosis. While, immune regulation is critical for the treatment of liver fibrosis induced by all types of etiologies.

### 2.2 Key Cellular Players

Significant advancements have been achieved in comprehending the pathophysiological processes underlying liver fibrosis, with the goal of creating more potent antifibrotic treatments. Initiated by factors we descripted above [6], early stages of liver fibrosis involve the disruption in energy metabolism, glucose/lipid metabolism, oxidative stress, endoplasmic reticulum stress (ERS), and immune response. These pathological changes can eventually lead to various types of cell death, including apoptosis, necrosis, pyroptosis, and ferroptosis, as well as cellular senescence in liver parenchymal cells, such as hepatocytes and cholangiocytes [20]. A series of DAMPs, such as high‐mobility group box 1 (HMGB1), mitochondrial DNA (mtDNA), and ATP, along with other fibrotic/inflammatory mediators and extracellular vehicles (EVs) released by these injured cells, can activate immune cells such as KCs and lymphocytes [21]. Cells activated by these DAMPs release inflammatory cytokines such as IL‐1β, TNF‐α, PDGF, and TGF‐β, along with chemokines including CCL2 and CXCL10, which further recruit peripheral monocytes and other immune cells to the site of injury [22]. These monocytes differentiate into inflammatory macrophage populations that aggravate liver fibrosis, further participating in tissue remodeling by secreting matrix metalloproteinases (MMPs). At the same time, the liver immune microenvironment is dominated by Th2 and Th17 responses, which further promotes inflammation and tissue fibrosis [23]. Moreover, TNF‐α, IL‐1β, PDGF, TGF‐β, IL‐4, and IL‐22 secreted by KCs and monocytes can directly activate HSCs via pathways such as TGF‐β/small mother against decapentaplegic (Smad), mitogen‐activated protein kinase (MAPK), and Hedgehog (Hh). Activated HSCs then transform into myofibroblasts that secrete excessive Collagen I and Collagen III, leading to an increase in ECM production. Hepatocytes, immune cells, and HSCs not only play critical roles in liver fibrosis, but also, as summarized in a previous review, facilitate the progression of liver fibrosis through the secretion of various EVs, establishing a complex intercellular communication network [21].

### 2.3 Core Signaling Pathways and Molecular Mechanisms

Core pathways driving fibrosis are summarized here. HSCs are the core fibrogenic cells, the mechanisms underlying their activation have been widely studied [8]. As described above, multiple cytokines are involved in HSC activation; among these, TGF‐β, the most potent fibrogenic cytokine, is released by several types of liver cells, including the activated HSCs itself. After binding to TGF‐β, the Type I receptor becomes phosphorylated, which activates SMAD proteins and promotes the transcription of Type I and Type III collagen. The activation of SMAD pathway also upregulated other profibrotic genes, including those encoding alpha‐smooth muscle actin (α‐SMA) and connective tissue growth factor [24]. Moreover, TGF‐β activates MAPK signaling pathways to promote HSC proliferation. Another factor, PDGF, can activate the specific tyrosine kinase receptor PDGF receptor‐β (PDGFRβ) on HSCs, initiate intracellular signal transduction, as well as regulate HSC proliferation and migration. Furthermore, hepatocytes produce VEGF to promotes HSC activation and proliferation, leading to increased production of ECM proteins and TGF‐β.

In addition, except for these ligands, the receptors on HSCs and other multiple signaling pathways in HSCs also regulate their activation. HSCs express Notch receptors on their surface, and binding to corresponding ligands allows the Notch intracellular domain to translocate into the nucleus and bind to RBP‐Jκ, thereby regulating the expression of downstream genes that activate HSCs, such as *ACTA2* and *COL1A1*. Wnt signaling exerts its effects through the activation of β‐catenin. By preventing β‐catenin degradation, the nuclear translocation of β‐catenin increases, which further promote the expression of genes involved in HSC activation. The canonical Hh pathway is also a conserved and highly complex signaling cascade. When Hh ligands bind to the Patched receptor, it activates the Smoothened signal transducer, which triggers the pathway and encourages the transcription factor GLI2 to accumulate in the nucleus. This drives the transcription of glioma‐associated oncogene homologue 1 (GLI1) and its target genes that involved in regulating HSC activation. The Hippo pathway effector YAP accumulates in the nucleus during the early activation of HSCs. Inhibition of YAP can prevent HSC activation and fibrosis, as well as reduce the expression of α‐SMA and Type I collagen [25].

### 2.4 ECM Dynamics

In a healthy liver, Type I, III, and V collagens are mainly located in the space of Disse, around the portal tracts, and along the central vein walls, while Type IV collagen is mostly present in the sinusoidal walls and helps form the vascular basement membrane. As fibrosis progresses pathologically, both portal fibroblasts and HSCs‐derived myofibroblasts continuously produce ECM, resulting in an increase of fibrillar collagen especially Types I and III collagen to replace areas where hepatocytes are lost. Alterations in the ECM also regulate HSC activation, with integrin signaling acting as a key mediator and forming a positive feedback loop. Additionally, growth factors such as PDGF, HGF, FGF, EGF, and VEGF are stored in ECM, which can further promote the proliferation of HSCs, thus accelerating the progression of liver fibrosis.

Understanding the mechanisms of ECM biosynthesis, deposition, and degradation may offer strategies for improving the management of fibrosis. For ECM biosynthesis, genes encoding Type I collagen, such as *COL1A1* and *COL1A2* are transcribed. Subsequently, La ribonucleoprotein domain family member 6 and miRNA29 are involved in the posttranscriptional modification of the transcripts of *COL1A1* and *COL1A2*. Posttranslational modifications are mediated by proteins such as glucose‐regulated protein 78 (GRP78), GRP94, and protein disulfide isomerase. Collagen maturation occurs extracellularly. Once procollagen is secreted into the extracellular space, its N‑ and C‑terminal peptides are cleaved by several proteases, which is achieved by bone morphogenetic protein 1 and certain members of the ADAMTS family, leading to the formation of mature collagen molecules [26]. These procollagen molecules then spontaneously aggregate, undergoing intramolecular and intermolecular crosslinking, with lysyl oxidase (LOX) catalyzing the formation of the final collagen fibrils. Due to the tightly packed helical structure of fibrillar collagens, most proteolytic enzymes are unable to degrade them efficiently. The degradation of these collagen primarily depends on MMPs [27]. However, the activity of MMPs is inhibited by tissue inhibitors of metalloproteinases (TIMPs), specifically the TIMP1–4 family. Typically, MMPs are downregulated, while TIMPs are upregulated in liver fibrosis.

Drugs targeting crucial genes involved in the biosynthesis, deposition, and degradation of ECM are currently under development for the treatment of liver fibrosis. Luangmonkong et al. provided a detailed review, indicating that most of the drug developments are in preclinical stages [28]. Notably, BMS‐986263 targeting HSP47 and simtuzumab targeting LOXL have entered clinical trials. BMS‐986263 has shown promising results in advanced fibrosis patients with good resistance to drug resistance [29]. In clinical trial, simtuzumab (an anti‐LOXL2 monoclonal antibody) has not demonstrated satisfactory antifibrotic efficacy and been halted in Phase II clinical trials, which is attributed to the difficulty of macromolecular antibodies to cross the ECM and occupy collagen‐bound LOXL2 [30].

## 3 Diagnostic and Assessment Strategies

At present, the gold standard for clinical diagnosis of liver fibrosis remains the invasive detective approach, liver biopsy, combined with histological scoring systems. However, it is difficult to detect the disease in the early stage due to the insidious nature of liver fibrosis. Therefore, identifying early liver fibrosis based on noninvasive methods is also a key part of clinical diagnosis. Here, we present the latest research progress.

### 3.1 Gold Standard: Liver Biopsy and Histological Scoring Systems

Liver biopsy is applied as the “gold standard” to assess the stage and degree of chronic liver injury and also applied to determine the cause of liver diseases, which is a well‐established technique that has been used in clinical for a long time [31]. Liver biopsies are typically conducted percutaneously, though alternative methods such as laparoscopic, transjugular, or endoscopic approaches may also be used. The selection of a particular technique depends on multiple factors, including chest‐wall thickness and the patient's clinical status, particularly regarding the presence of thrombocytopenia or coagulopathy [32]. After liver tissue is obtained via liver biopsy, pathological testing is conducted to evaluate the degree and pattern of inflammation, steatosis, and fibrosis. As liver biopsy samples lack histological controls, the reproducibility of liver biopsy interpretation is of great importance. Additionally, most patients require multiple sequential histological tests as their liver disease progresses. To facilitate comparisons across different studies and assess changes during therapeutic trials, pathologists have developed categorical scoring systems to grade inflammation and steatosis, as well as stage fibrosis.

The Ishak score and Metavir score are the most internationally and widely used scoring systems for grading inflammatory necrosis and staging fibrosis in liver tissue of patients with viral hepatitis. Specifically, as described in 1998, the Ishak system, which is most commonly used for fibrosis staging in patients with various types of hepatitis, employs a scale ranging from F0 to F6 [33]. By increasing the number of classes for fibrosis staging, this system significantly enhances the accuracy of distinguishing the stage of fibrosis. The Ishak system is mostly used in studies requiring detailed fibrosis grading (e.g., drug development), but it is less frequently employed in routine clinical diagnosis. The Metavir scoring system was developed by a French research team specifically for chronic hepatitis C. As documented in 1994, it is most frequently used for fibrosis staging in patients with various types of hepatitis, with a scale ranging from F0 to F4 [34]. It is now regarded as the gold standard in global liver disease guidelines and is widely adopted, especially in the evaluation of chronic viral hepatitis and MASLD. Physicians generally restrict liver biopsy to patients having specific symptoms because of the attendant risks to skin and liver tissue. Moreover, sampling error and interobserver variation can reduce diagnostic reliability. A single liver biopsy sample may fail to capture heterogeneously distributed fibrotic lesions, leading to potentially unrepresentative results.

### 3.2 Noninvasive Tests

Early fibrosis is still reversible, but only if it is caught and treated appropriately. Given the nonsymptomatic nature of early‐stage liver fibrosis, invasive procedures such as liver biopsy are not optimal for its diagnosis. Therefore, the development of innovative noninvasive approaches for the evaluation and diagnosis of early‐stage liver fibrosis is crucial. Compared with liver biopsy, noninvasive diagnostic tools are safer, better tolerated, and more acceptable to patients. Additionally, noninvasive techniques provide feedback during therapy, enabling doctors to promptly adjust treatments.

Advancements in technology have led to the investigation of several noninvasive techniques, which fall into two categories: imaging‐based methods and serum‐based markers. Imaging‐based methods include ultrasound, magnetic resonance elastography (MRE), and elastography. Notably, vibration‐controlled transient elastography and MRE were applied for the determination of liver stiffness (LS) in patients with liver fibrosis, whose accuracy is higher than that of present serum markers [35]. LS measured through elastography technology has emerged as a valuable indicator of fibrosis burden in clinical efficacy evaluations of various small molecules. For example, two‐dimensional shear wave elastography has been developed recently and used in clinical practice [36]. Noninvasive blood tests can be divided into two categories: indirect markers and direct markers. Indirect markers include the aspartate aminotransferase to platelet ratio index, fibrosis‐4 index, and NAFLD fibrosis score. Direct markers include the enhanced liver fibrosis score, FibroTest, and FibroMete [37]. An increasing number of single serological markers have been identified. For example, plasma Follistatin‐like protein 1, PRO‐C3, Type IV Collagen 7S, and thrombospondin‐2 can accurately identify advanced liver fibrosis [38, 39, 40, 41]. However, most of these biomarkers still need rigorous validation in large‐scale multicenter cohorts before they can be reliably translated into clinical use. Furthermore, many of these biomarkers may also serve as potential therapeutic targets.

## 4 Etiological Treatment (Foundation of Therapy)

As we mentioned, liver fibrosis triggered by various etiologies, and currently, the therapeutic strategies focused on etiological treatment, which aims to prevent the disease by targeting its root drivers. For different etiologies, the characteristics vary, and the targeted therapeutic approaches also differ accordingly. For patients with MASLD, MASH, PBC, and PSC, targeted regulation of metabolic disorders, including lipid, glucose, and bile acid metabolism, is the current main approach. Moreover, reducing the number of viruses or inhibiting the immune cascade response triggered by viral infection is the treatment strategy for hepatitis C virus (HCV) and HBV‐related liver fibrosis. Relevant preclinical researches and clinical trials focusing on these mechanisms are comprehensively discussed in the present section.

### 4.1 Metabolic Regulator

**FIGURE 1:**
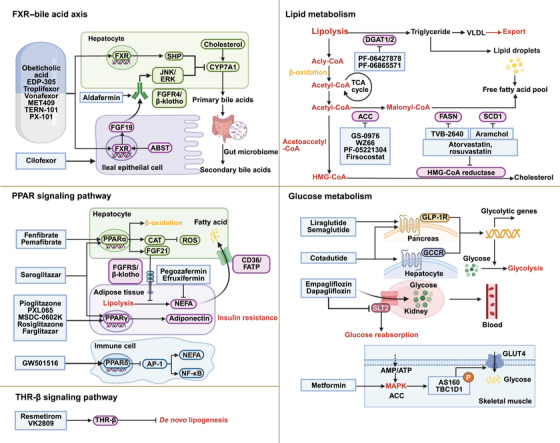
Regulating bile acid, lipid and glucose metabolisms have emerged as promising treatment strategies for liver fibrosis. FXR agonists and FGF19 analogs target the FXR–bile acid axis to reduce liver fibrosis, while PPAR agonists and FGF21 analogs focus on PPAR‐mediated lipid metabolism. THR‐β agonists improve liver fibrosis treatment by targeting THR‐β‐mediated lipid metabolism. In addition, the inhibitors of ACC, FASN, SCD1, DGATs, and HMG‐CoA reductase show potential in treating liver fibrosis by targeting key enzymes in hepatic lipid metabolism. GLP‐1R agonists, GCGR agonists, AMPK activators, and SGLT2 inhibitors have also been shown to alleviate liver fibrosis by targeting glucose metabolism.

For liver fibrosis induced by MASLD and MASH, current etiological treatments primarily focus on targeting the key pathological mechanisms underlying disease progression. Lipotoxicity is closely linked to the development of fibrosis in these metabolic‐associated liver diseases. Moreover, glucose metabolism dysfunction (particularly insulin resistance) drives fibrosis progression. Additionally, dysregulated bile acid homeostasis characterized by impaired bile acid efflux is a common feature of such chronic liver diseases, contributing to fibrotic changes. Thus, for liver fibrosis induced by MASLD, MASH, and PBC, current etiological treatment approaches primarily focus on regulating lipid metabolism, glucose metabolism, and bile acid metabolism (Figure 1). Herein, we have summarized the chemical compounds with potential clinical utility that target lipid metabolism, glucose metabolism, and bile acid metabolism, as well as the traditional Chinese medicines targeting bile acid metabolism for the treatment of liver fibrosis.

#### 4.1.1 Chemical Compounds Targeting Peroxisome Proliferator‐Activated Receptor Mediated‐Lipid Metabolism

Peroxisome proliferator‐activated receptor (PPAR), a member of the nuclear hormone receptor superfamily, acts as a ligand‐activated transcription factor through heterodimer formation with the retinoid X receptor and binding to specific DNA regions in target genes. Given its regulatory roles in lipid metabolism, glucose balance, and inflammatory responses, PPAR represents an important therapeutic target for alleviating liver fibrosis driven by metabolic disturbances and inflammation. There are three subtypes of PPAR: PPARα, PPARδ, and PPARγ, each with different tissue distribution and target gene specificity.

PPARα is predominantly expressed in liver hepatocytes, where it regulates the oxidation of FAs and energy metabolism by modulating the expression of lipoprotein lipase, apolipoprotein genes, and genes involved in FA transport and oxidation. As a selective PPARα modulator, fenofibrate mitigated dyslipidemia induced by acetyl‐CoA carboxylase (ACC) inhibition, leading to greater improvement in liver fibrosis in MASH patients when used in combination with farnesoid X receptor (FXR) agonist, cilofexor, and firsocostat (GS‐0976, ACC inhibitor). Notably, pemafibrate and fenofibrate were reported to ameliorate HSC activation and hepatic stiffness without significantly reducing liver lipid content in individuals with MASLD/MASH [42]. Notably, the combination of fenofibrate and pentoxifylline showed greater benefits than fenofibrate monotherapy on biochemical parameters and LS in patients with MASLD, providing more therapeutic choices for liver fibrosis. Based on the above evidence, pemafibrate and fenofibrate have become candidates for combination therapy with lipid‐lowering drugs.

PPARγ activation promotes glucose utilization and reduces gluconeogenesis to regulate blood glucose levels. It also enhances hepatic glucose uptake, resulting in the improvement of insulin sensitivity for patients with liver fibrosis. Pioglitazone, an oral PPARγ agonist primarily used for T2DM, has shown potential therapeutic effects in liver fibrosis. Patients with MASH and prediabetes or T2DM who took pioglitazone for 18 months showed significant improvement in MASLD/liver fibrosis scores. Another study added that pioglitazone showed greater improvement in liver fibrosis and insulin sensitivity in MASH patients with T2DM compared with those without T2DM [43]. However, the clinical utility of pioglitazone is limited by PPARγ‐related side effects, such as edema and weight gain. PXL065 is a novel deuterium‐stabilized R‐pioglitazone enantiomer that not only preserved the efficacy of pioglitazone in MASH patients with a reduced potential for PPARγ‐driven side effects, but also demonstrated higher bioavailability [44]. MSDC‐0602K, a second‐generation insulin synergist, has shown effectiveness in reducing side effects linked to first‐generation insulin sensitizers in clinical trials for MASH patients by targeting the mitochondrial pyruvate carrier and minimizing direct interaction with the transcription factor PPARγ [45]. Furthermore, ongoing research includes the development of PPARγ agonists such as rosiglitazone and farglitazar (NCT00244751).

PPARδ is extensively distributed throughout the body and plays a role in lipid metabolism and insulin sensitivity. The PPARδ agonist GW501516 has been shown to decrease body weight in mice with steatohepatitis. Nevertheless, its impact on liver histology may not be significant, potentially attributable to the brief duration of treatment [46]. Another PPARδ agonist, seladelpar, demonstrated a favorable safety profile and clinically significant efficacy in adults with PBC, positioning it as a promising second‐line treatment for PBC [47].

Some dual PPAR agonists or pan‐PPAR agonists have shown better therapeutic effects in addition to the selective PPAR agonists mentioned above. Saroglitazar, a novel PPARα/γ agonist, rectifies lipid‐mediated oxidative stress, inflammation, and impaired mitochondrial biogenesis in choline‐deficient, high‐fat diet (HFD)‐induced MASH and carbon tetrachloride (CCl_4_)‐induced fibrosis mouse models. Notably, the reduction in total MASLD activity score observed with saroglitazar was significantly greater than with pioglitazone and fenofibrate in the MASH model [48]. Additionally, saroglitazar was observed to reduce alanine aminotransferase (ALT) levels and liver fat content and improve insulin resistance and thermogenic dyslipidemia in patients with MASLD in a Phase II clinical trial [49]. Currently, the Drug Controller General of India has approved saroglitazar for treating MASH. This approval is expected to significantly reduce the incidence and improve the outcomes for patient with liver fibrosis, particularly in hepatitis patients. Another dual PPARα/δ agonist, elafibranor, has garnered attention for its potential in treating patients with MASH. Results from a 1‐year Phase II clinical trial showed that a higher proportion of MASH patients treated with elafibranor did not experience worsening fibrosis compared with those who received a placebo. Furthermore, significant reductions in liver enzymes and systemic markers of inflammation were observed in the elafibranor group [50]. Elafibranor is the third drug approved for the treatment of PBC and the first new drug approved in the past decade for this indication. It is also the first PPAR agonist approved for the treatment of PBC. Among the new drugs in development, lanifibranor, which simultaneously activates all three PPAR isoforms, is expected to be the first oral drug approved by the US FDA to treat MASH, with the potential to inhibit fibrosis. In contrast to selective PPARα (fenofibrate), PPARγ (pioglitazone), and PPARδ (GW501516) agonists, pan‐PPAR agonist lanifibranor combines their beneficial effects to ameliorate liver fibrosis in various animal models induced by thioacetamide treatment, choline deficiency, amino acid‐defined HFD, western diet, and CCl_4_ [46, 51]. Most encouragingly, lanifibranor treatment in Phase II trials resulted in significant improvements in levels of inflammation and fibrosis in MASH, particularly in highly active MASH patients with moderate or advanced liver fibrosis [52]. These positive results supported the further advancement of lanifibranor into Phase III trials (NCT04849728), with the aim of evaluating its potential to slow down or reverse the disease progression and improve liver fibrosis in patients with MASH and fibrosis stages F2/3.

FGF21, a key metabolic messenger mainly controlled by PPARs in the liver, regulates glucose–lipid homeostasis through β‐klotho activation of FGF receptors. Recently, several prolonged half‐life FGF21 analogs have been in clinical development for reversing fibrosis in patients with metabolic disorders. For instance, pegbelfermin extends its half‐life via conjugating PEG molecules to the recombinant FGF21 protein, notably reducing the fibrosis score by more than 1 point in patients with Stage 3 (bridging) fibrosis secondary to MASH in Phase IIb clinical trials [53]. As a bivalent FGF21 analog, efruxifermin conjugates the Fc domain of human IgG to the recombinant FGF21, thereby achieving a longer half‐life and enhanced liver targeting ability compared with pegbelfermin. Functionally, efruxifermin exhibited a favorable safety and tolerability profile and demonstrated a significant reduction in liver fat content and regression of fibrosis in patients with phenotypically defined MASH in two Phase IIa trials [54, 55]. Efruxifermin also significantly reduced hepatic fat and liver injury biomarkers in patients with MAFLD, while improving glucose and lipid metabolism as well as liver fibrosis in a Phase IIb trial [56]. In this report, there were no reports of drug‐induced liver injury or fatalities, suggesting a favorable clinical application potential. However, unfortunately, in the cohort of patients with compensated cirrhosis secondary to MASH, efruxifermin did not achieve a statistically significant improvement in fibrosis status following 36 weeks of treatment [57].

#### 4.1.2 Chemical Compounds Targeting Thyroid Hormone Receptor‐β‐Mediated Lipid Metabolism

Thyroid hormones directly regulate lipogenesis, FA β‐oxidation, cholesterol synthesis, and the reverse cholesterol transport pathway by acting at the transcriptional, posttranslational, and autophagic levels [58]. Due to their potential beneficial effects on lipid metabolism, thyroid hormones can improve metabolic diseases of the liver. Recently, thyroid hormone receptors (THRs) (THR‐β is the main receptor subtype in the liver) are reported to exert beneficial effects on liver fibrosis by increasing hepatic fat metabolism and reducing lipotoxicity. The THR‐β agonist resmetirom can improve liver metabolic parameters, reduce MASLD activity scores, and decrease liver fibrosis in MASH mice [59, 60]. As expected, the improved effects of resmetirom on liver fat and MASLD activity scores without worsening liver fibrosis were also demonstrated in MASH patients in a Phase II clinical trial [61, 62]. Notably, the Phase III clinical study (NCT05500222) of resmetirom achieved positive results for MASH patients, meeting primary endpoints including relieving MASH symptoms, reducing MASLD activity score (≥2 points) without worsening liver fibrosis or improving liver fibrosis by at least one point without worsening liver fibrosis. Following this success, resmetirom has obtained approval from the US FDA for patients with MASH, marking a significant milestone as the first therapy for this condition. Similarly, the liver‐targeted THR‐β agonist VK2809 prevented the development of cirrhosis in mice by simultaneously restoring autophagy, mitochondrial biogenesis, and FA β‐oxidation [63], showing positive efficacy in MASH patients with liver fibrosis in a Phase IIb clinical trial (NCT04173065).

#### 4.1.3 Chemical Compounds Targeting Other Critical Enzymes Involved in Hepatic Lipid Metabolism

Several rate‐limiting enzymes are involved in hepatic lipid metabolism, including reductase ACC, FA synthase, stearoyl‐CoA desaturase 1, diacylglycerol acyltransferases (DGATs), and 3‐hydroxy‐3‐methylglutaryl‐coenzyme A (HMG‐CoA). Modulating the activities of these enzymes can alleviate fibrosis, either by reducing lipid synthesis at the source or promoting downstream breakdown, thereby reducing lipotoxic injury in the liver.

The FA synthase inhibitor, TVB‐2640, significantly improved hepatic steatosis in MASH patients after 12 weeks, accompanied by reduced serum markers of liver fibrosis or gene expression regulating fibrosis [64]. Several clinical trials involving patients with MAFLD revealed that the ACC inhibitor, firsocostat, significantly reduced liver fat content and fibrosis biomarkers in MAFLD patients [65, 66]. In addition to monotherapy, the combination therapies involving firsocostat (an ACC inhibitor) and cilofexor (an FXR agonist) showed promise in treating bridging fibrosis and cirrhosis in MASH patients in a Phase II trial [67]. The combination of PF‐05221304 (an ACC inhibitor) with PF‐06865571 (a DGAT2 inhibitor), rosuvastatin (an HMG‐CoA reductase inhibitor) with aldafermin (an FGF19 analogue) or ezetimibe (a niemann‐pick C1‐like 1 inhibitor) all showed a favorable overall lipid profile and improved fibrotic situation in patients with fibrotic injuries [68, 69, 70]. However, the monotherapy of firsocostat or the combination of firsocostat and cilofexor still have some limitations, such as increasing serum levels of triglycerides (TGs). Fenofibrate alleviates hypertriglyceridemia in MASH patients treated with firsocostat and cilofexor. Based on the good bioavailability and efficacy of firsocostat, introducing more drug combinations to address its side effect of increasing serum TG levels may have better clinical application prospects.

Consistently, another ACC inhibitor WZ66 was found to inhibit the activation of KCs and HSCs in obese mice [71]. Although high doses of aramchol (a partial inhibitor of hepatic stearoyl‐CoA desaturase 1) did not achieve the expected level of hepatic lipid reduction within 52 weeks, it improved fibrosis by ≥1 stage in patients with MASH in a Phase IIb clinical trial [72]. Moreover, the clinical evaluation of aramchol's efficacy in treating patients with Stage 2–3 fibrotic MASH is currently in Phase III trials (NCT04104321). On the other hand, the DGAT2 inhibitor PF‐06427878 has shown promising results in reducing hepatic lipid accumulation, inflammatory histological features, HSC activation, and ECM deposition in the western diet‐induced MASLD and the STAM MASH–HCC mouse models, as well as modifying key liver biomarkers and liver fat in healthy adults [73]. However, clinical studies on PF‐06427878 for the treatment of MASH have been discontinued, while the efficacy of the follow‐up product PF‐06865571, derived from PF‐06427878, is now being evaluated in Phase II clinical trials for treating MASH patients with liver fibrosis. In addition to its lipid‐lowering activity, the HMG‐CoA inhibitor atorvastatin has been shown to modulate the expression of miR‐21 and miR‐122, thus downregulating TGF‐β, which can halt the progression of liver fibrosis [74].

#### 4.1.4 Chemical Compounds Targeting Glucose Metabolism

Glucagon‐like peptide‐1 (GLP‐1) and gastric inhibitory polypeptide (GIP) are gut‐derived hormones that are released within minutes of nutrient ingestion. These hormones enhance glucose‐dependent insulin secretion through activation of the pancreatic GLP‐1 receptor (GLP‐1R) and the GIP receptor (GIPR), respectively. Emerging experimental research has demonstrated that GLP‐1R and GIPR agonists exert an antifibrotic effect by influencing insulin resistance and reducing steatosis. The decrease in de novo adipogenesis and the increase in FA oxidation are underlying mechanisms. Those findings have been successfully translated into clinical studies. A dual GIP and GLP‐1 receptor agonist, tirzepatide has been shown to alleviate MASH and reduce the level of fibrosis biomarkers in patients with Type 2 diabetes [75]. Compared with multitarget agonists, more research has focused on GLP‐1R‐specific agonists. Recently, semaglutide, approved by the US FDA in 2017 for the treating Type 2 diabetes, has demonstrated significant improvements in liver fibrosis and shown potential for the treatment of MASLD in a Phase III clinical trial [76]. The long‐term efficacy of the GLP‐1R agonist, liraglutide, led to histological resolution of MASH and arrest of liver fibrosis in MASH patients in Phase II studies [77]. Animal studies have shown a potentially enhanced effect when it was combined with elafibranor (PPARα/δ agonist) or bempedoic acid (ATP citrate lyase inhibitor) [78, 79]. Although another GLP‐1R agonist called semaglutide did not show significant improvement in liver fibrosis as a monotherapy in fibrotic patients caused by metabolic disorders [80, 81], there were greater improvements observed in liver steatosis, liver biochemistry, and noninvasive tests of fibrosis when combined with cilofexor (FXR agonist) and firsocostat (ACC inhibitor) [82]. Unlike the above two drugs, cotadutide is a dual agonist, activating both the GLP‐1R and glucagon receptors, which, notably, was more effective than either liraglutide (GLP‐1R agonist) or FXR agonist, obeticholic acid (OCA) in treating HFD‐induced liver fibrosis in a preclinical experiment [83]. Similarly, another dual GLP‐1‐glucagon receptor agonist, pemvidutide, attained the primary endpoint of MASH resolution with stable fibrosis status at 24 weeks; however, it did not achieve the parallel primary endpoint of fibrosis amelioration without MASH progression at this same assessment timepoint [84]. In addition to GLP‐1 agonists, dapagliflozin, a sodium‐glucose cotransporter 2 (SGLT2) inhibitor, demonstrated efficacy in attenuating the progressive deterioration of liver fibrosis among MASH patients in clinical trial [85]. Interestingly, a study suggests that the combination of exenatide (GLP‐1R agonist) and dapagliflozin was more successful in improving markers of hepatic steatosis and fibrosis in patients with T2DM compared with either drug used alone [86].

The AMP‐activated protein kinase (AMPK) activator metformin and sodium‐dependent glucose cotransporter 2 inhibitors empagliflozin and dapagliflozin, approved for the treatment of T2DM and insulin resistance, have recently shown promise in halting or reversing MASLD‐related fibrosis, highlighting the inseparable relationship between the improvement of MASLD‐related fibrosis and glucose metabolism. Preclinical studies have indicated that both metformin and empagliflozin inhibited the TGF‐β pathway, thereby improving fibrosis caused by chemical toxins or unhealthy diets [87, 88]. At the same time, the combination of metformin and empagliflozin markedly activated AMPK, inhibited p38 MAPKα and extracellular regulated protein kinases (ERK) activities, offering a promising therapeutic strategy for the prevention and treatment of fibrosis in obese patients [89]. In addition, metformin was reported to prevent liver tumorigenesis by attenuating fibrosis in a transgenic mouse model of HCC [90]. These positive results were further confirmed in clinical trials. Long‐term use of metformin improves clinical outcomes in diabetic patients with bridging liver fibrosis or compensated cirrhosis [91], while the sodium‐dependent glucose cotransporter 2 inhibitor dapagliflozin reduced liver fat content and attenuated liver fibrosis in individuals suffering from T2DM and MASLD with severe liver fibrosis [92]. As a key target, relevant agonists targeting AMPK have been continuously developed. More encouragingly, the direct AMPK activator PXL770 has entered Phase II clinical trials, which has been shown to reduce HbA1c levels and insulin resistance in patients with MAFLD [93]. The clinical success of PXL770 may be attributed to its moderate potency; compared with potent AMPK agonists, it can activate AMPK in a more physiological manner.

#### 4.1.5 Chemical Compounds and Traditional Chinese Medicine Targeting the FXR–Bile Acid Axis

When activated, FXR, a nuclear receptor activated by bile acid, maintains the homeostatic balance of TGs in the liver and circulating blood by inhibiting TG synthesis, promoting TG catabolism, and regulating lipoprotein secretion. In addition to regulating metabolism, FXR activation may aid in the treatment of liver fibrosis by inhibiting TGF‐β to suppress HSC activation, suppressing NOD‐like receptor family pyrin domain containing 3 (NLRP3) and reducing the release of proinflammatory cytokines, inhibiting CYP7A1 to regulate bile acid metabolism, upregulating FGF19 and FGF21 to alleviate hepatic metabolic stress, and enhancing peroxisome PPARα to promote FA catabolism [94]. Activating this target may influence multiple stages in the liver fibrosis progression that we have mentioned, making it a crucial therapeutic target. Recently, a variety of high‐affinity ligands targeting FXR have been developed as drug candidates for the treatment of liver fibrosis, categorized primarily as bile acid analogs and non‐bile acid analogs.

OCA, with the chemical structure of 6α‐ethylodeoxycholic acid, could activate FXRs distributed in the gut and liver. Currently, OCA is the only FXR agonist approved by both the US FDA and the European Medicines Agency as a second‐line drug for the treatment of PBC [95, 96]. The clinical trial of OCA for liver fibrosis in individuals with PSC also showed promising results, including a notable decrease in serum alkaline phosphatase levels that persisted over a 2‐year long‐term safety extension period [97]. Similarly, in the Phase III clinical trial, after receiving OCA treatment for 18 months, MASH patients with F2–F3 stage fibrosis demonstrated improvement (≥1 stage) or no worsening in fibrosis [98]. However, the therapeutic potential of OCA is limited by side effects including pruritus, dyslipidemia, and possible hepatotoxicity, which are believed to be related to the full agonistic effects of FXR due to its bile acid‐like structure. An experimental study proposed a novel approach of combining lower doses of OCA with the apoptosis inhibitor IDN‐6556, which effectively inhibited HSC activation and proliferation while minimized potential side effects of OCA and maintained bile acid homeostasis [99].

Non‐bile acid FXR agonists are currently being developed to address the clinical limitations caused by the side effects of OCA. Unlike OCA, these non‐bile acid agonists are expected to exhibit distinct pharmacokinetic, efficacy, and safety profiles. Among these new non‐bile acid FXR agonists, EDP‐305 stands out as a promising candidate due to its steroidal structure. EDP‐305 not only outperformed OCA in improving liver fibrosis in murine biliary and metabolic models of liver disease [100], but also reduced magnetic resonance imaging‐PDFF in patients with fibrotic MASH in a Phase II clinical trial [101]. Despite showing efficacy through FXR in the high‐dose group of EDP‐305, 47% of patients experienced severe pruritus, and 21% of them discontinued the treatment due to this side effect. Cilofexor (GS‐9674) has been shown to activate FXR primarily in the gut without enterohepatic circulation, which reduces the adverse effects of systemic FXR activation. For noncirrhotic patients with PSC or MASH in a Phase II clinical trial, patients treated with cilofexor experienced significant reductions in serum markers of fibrosis, as well as improvements in liver histological manifestations and serum biochemical indicators [102, 103]. To our surprise, MASH patients but not PSC patients still experienced moderate to severe pruritus after taking cilofexor, which warrants further investigation. Unfortunately, the Phase III trials (NCT03890120) evaluating the effect of cilofexor on PSC patients with fibrosis Stages F0–F3 were terminated due to the low likelihood of improving liver fibrosis scores. Similarly, in participants with noncirrhotic PSC, cilofexor showed no significant difference in fibrosis progression rates when compared with placebo and experienced severe pruritus in a Phase III clinical trial [104]. Tropifexor, another novel and potent FXR agonist that attenuated steatosis, inflammation, and fibrosis in a mouse model of MASH [105], has entered Phase II clinical trials for patients with MASH and PBC (NCT02913105). In the Phase IIb TANDEM trial, tropifexor has shown promising results in treating metabolic dysfunction‐associated liver fibrosis, including at least a 1‐point improvement in fibrosis stage or steatohepatitis resolution without worsening fibrosis [106]. In addition, as a second‐generation nonsteroidal and non‐bile acid FXR agonist, vonafexor (EYP001) has demonstrated effectiveness in lowering liver fat, improving imaging biomarkers related to liver enzymes and fibrotic lipoid hepatitis, and potentially enhancing renal function for patients with suspected fibrotic MASH [107]. Although vonafexor can also improve chronic hepatitis B, it still presents gastrointestinal side effects and pruritus side effects similar to those of bile acid drugs [108]. Another non‐bile acid FXR agonist, named MET409, was identified following an iterative structure–activity relationship analysis of over 2500 non‐bile acid FXR agonists. In a 12‐week, randomized, placebo‐controlled study, MET409 demonstrated encouraging results, including a class‐leading liver fat reduction and liver enzyme improvement accompanied by differentiated pruritus and LDL‐C profile compared with other FXR agonists [109]. However, pruritus remains an unavoidable side effect. MET642, derived from the same chemical scaffold as MET409, has shown greater potency and distinct pharmaceutical properties than MET409. Currently, the Phase II clinical trial (NCT04773964) evaluating MET642 in MASH patients has been completed. Furthermore, FXR agonists such as TERN.101 (LY2562175) and PX‐102 are under active investigation to prevent and reverse the progression of MASH [110].

Although targeting FXR demonstrated ideal clinical efficacy, both bile acid and non‐bile acid agonists generally have pruritus as a side effect, which limits their clinical application. Recent studies have shown that the pruritus side effect caused by OCA is due to its activation of the hX4 receptor [111]. By making precise structural modifications to OCA and removing the key group responsible for activating hX4, a novel lead compound, C7, was created. This compound lost its ability to activate hX4 and induce pruritus, but retained its FXR activation ability, which is responsible for its therapeutic effects, demonstrating tremendous clinical potential.

Ursodeoxycholic acid (UDCA), a hydrophilic bile acid, is widely used in the treatment of cholestatic liver diseases, including PBC, intrahepatic cholestasis of pregnancy, and cystic fibrosis with hepatic involvement. In contrast to the action mechanism of FXR agonists, UDCA ameliorated liver fibrosis by stimulating bile secretion from bicarbonate‐rich bile ducts and protecting hepatic tissues from damage caused by toxic bile acids [112]. Notably, UDCA was also found to have an inhibitory effect on FXR targets, affecting cholesterol and bile acid synthesis in morbidly obese MASLD patients, leading to the accumulation of neutral lipids in the liver and visceral white adipose tissue [113]. Despite not being recommended for treatment of MASLD due to potential exacerbation risks, UDCA demonstrated antifibrotic therapeutic effects in a CCl_4_‐induced rat model by inhibiting collagen production and viability of activated HSCs through the suppression of autophagy [114].

FGF19 is an intestinal hormone released from the terminal ileum following intestinal FXR activation, which further binds the liver receptor homolog 1 to inhibit TG synthesis. However, endogenous FGF19 is unsuitable as a medication for clinical treatment due to the risk of HCC occurrence caused by FGF19 overexpression in the liver. To address this concern, several FGF19 analogs have been designed to mitigate the risk of carcinogenesis while exert similar antifibrotic actions by targeting bile acid and glucolipid metabolism. Aldafermin (NGM282) is an engineered analogue of FGF19. In a clinical trial, aldafermin improved histological characteristics of individuals with biopsy‐confirmed MASH within a 12‐week period. This improvement was indicated by the decrease in liver fat content, nonalcoholic fatty liver disease activity score, and fibrosis scores, along with enhancements in noninvasive imaging and serum markers [115]. Regrettably, although there were some favorable outcomes observed in secondary endpoints and acceptable gastrointestinal side effects, the Phase IIb clinical trial demonstrated only a minimal improvement in stage F2–F3 fibrosis following treatment with aldafermin [116]. The finding may impact the future design proposal of MASH trials and the clinical utility of aldafermin.

Traditional Chinese medicine formulas that regulate bile acid have also shown promising antifibrotic effects. Research has demonstrated that the mechanism of Da‐Chai‐Hu decoction and Qingzhi‐Tiaogan decoction inhibited BA synthesis, secretion, and enhanced BA efflux to ameliorate liver fibrosis in vivo. The specific mechanisms by which traditional Chinese medicine regulates this process have been comprehensively discussed in another review [117].

### 4.2 Antiviral Modulators

Importantly, in the treatment strategy of HCV and HBV‐related liver fibrosis, either reducing the number of viruses or inhibiting the immune cascade response triggered by viral infection of liver cells can reverse the consequences of liver fibrosis. For instance, many antiviral drugs, such as sofosbuvir, tenofovir disoproxil fumarate, and entecavir, mitigate virus‐induced liver fibrosis by inhibiting viral replication. Furthermore, compared with antiviral monotherapy, the combination of the herbal medicine Biejia‐Ruangan and entecavir that resulted in a significantly higher rate of fibrosis regression and a reduced risk of HCC in patients with chronic hepatitis B (CHB) [118, 119], which provides a new approach for the treatment of fibrosis. However, whether these antiviral compounds have regulatory effects on other stages of the fibrosis process remains unknown. Other clinical trials revealed that tenofovir disoproxil fumarate reduced the risk of liver fibrosis advancement in those suffering from CHB [120], but did not improve liver fibrosis levels in coinfected human immunodeficiency virus–HBV patients [121]. Therefore, extrapolating their use in the treatment of liver fibrosis caused by other etiologies should be approached with caution.

As we described above, in the early stages of viral infection or in cases where the infection is potentially eradicated, strengthening the immune system to recognize and eliminate HBV/HCV viruses can effectively prevent the diseases progression. However, when the virus has established a chronic infection, the immune system is unable to completely clear the virus. Instead, it continues to attack both infected hepatocytes and inadvertently damaging healthy tissue, leading to long‐term chronic inflammation. This abnormal inflammation is the key driver of fibrosis. Thus, long‐term administration with tacrolimus, azathioprine, and short‐term prednisolone can help slow the progression of fibrosis and reduce portal hypertension and decompensation in successive liver transplant recipients with HCV cirrhosis [122]. Interestingly, immunosuppression with a mammalian target of rapamycin (mTOR) inhibitor everolimus was found to be more effective in reducing fibrosis progression in liver transplant patients with HCV infection compared with calcineurin inhibitor [123]. On the other hand, half of the patients with post‐liver transplant HCV and severe fibrosis experienced a regression of the fibrosis stage and a long‐term improvement in liver function after the successful completion of sofosbuvir‐based therapy [124].

## 5 Targeted Antifibrotic Therapies (Mechanism‐Based Therapies)

No matter which etiological factor leading to liver fibrosis, once liver fibrosis occurs and progresses to the advanced stage, it will encounter many common damaging processes that exacerbate its progression, including oxidative stress, programmed cell death, excessive immune responses, and the activation of HSCs. In recent years, therapeutic strategies targeting the above processes have received increasing attention, and the relevant therapeutic approaches may have a universal therapeutic effect on liver fibrosis of different etiologies.

### 5.1 Medicines Targeting Oxidative Stress

**FIGURE 2:**
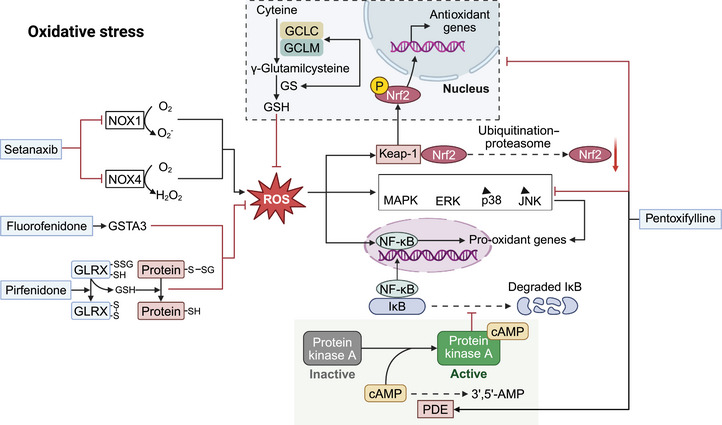
Inhibiting oxidative stress is regarded as a treatment strategy against liver fibrosis. Liver fibrosis often arises from an imbalance in oxidative stress due to damage to hepatocytes. Setanaxib has shown effectiveness in reducing the production of ROS by specifically targeting NOX1/4, thus contributing to the improvement of liver fibrosis. Moreover, fluorofenidone counteracted oxidative stress and alleviated liver fibrosis by reinstating the expression of reduced GSTA3. Pentoxifylline inhibited oxidative stress and ameliorated liver fibrosis through the regulation of the Nrf‐2/Keap‐1 and NF‐κB p65/p38–MAPK signaling pathways. Additionally, pirfenidone can reverse PSSG in a GLRX‐dependent manner, leading to the inhibition of oxidative stress and ultimately aiding in the improvement of liver fibrosis.

Herein, we summarized the chemical compounds with potential clinical utility, preclinical natural compounds and traditional Chinese medicines that target oxidative stress. First, we focus on small compounds that have entered clinical trials and exert antifibrotic effects by targeting oxidative stress. The initiation of liver fibrosis may not always result from the direct activation of HSCs, but rather from an imbalance of oxidative stress and excessive immune response induced by hepatocyte damage. ROS generated during oxidative stress plays a role in triggering lipid peroxidation (LPO), leading to the production of 4‐hydroxynonenal and malondialdehyde. These molecules, along with ROS, activate redox‐sensitive signaling pathways that promote the activation of HSCs, including the Nrf‐2/Keap1, MAPK, and Wnt/β‐catenin pathways. Therefore, targeting the generation of ROS and the activity of redox‐sensitive signaling pathways appears to be beneficial in suppressing liver fibrosis (Figure 2). The NADPH oxidase (NOX) family catalyzes the controlled production of reactive oxygen species. Among the NOX family, NOX1 and NOX4 can promote hepatic oxidative stress and LPO, thereby promoting liver fibrosis. Preclinical and Phase II clinical studies have proven that setanaxib (GKT137831), a highly selective NOX1/4 inhibitor, exerted a significant beneficial effect on immune response and fibrosis resulting from dysregulated lipid or bile acid metabolism [125, 126]. A Phase III clinical trial (NCT05014672) is currently underway to evaluate the effectiveness of setanaxib in the treatment of PBC and LS. The antioxidant protease glutathione S‐transferase A3 is being investigated as a possible signaling molecule that mediates the regulation of MAPK and Wnt/β‐catenin pathways by ROS or 4‐hydroxynonenal. Several advances confirmed that fluorofenidone (AKF‐PD) reduced ROS accumulation in HSCs and lowered LPO levels by restoring the decreased expression of glutathione S‐transferase A3 in the fibrosis model, thereby exhibiting an antifibrotic effect [127]. Redox‐sensitive protein cytoglobin can protect HSCs from oxidative stress, inhibiting their differentiation into MF‐like phenotypes. Pentoxifylline, a nonselective phosphodiesterase inhibitor, has demonstrated antifibrotic properties by elevating cytoglobin levels and modulating the Nrf‐2/Keap1 and nuclear factor kappa‐B (NF‐κB) p65/p38–MAPK signaling pathways [128]. Meanwhile, in mice with MASH, pentoxifylline was found to rescue the antifibrotic effect of OCA by inhibiting LPO [129]. Moreover, in individuals with MASLD, the coadministration of pentoxifylline with fenofibrate improved hyaluronic acid and TGF‐β levels, inflammatory pathways, and LS [130]. During oxidative stress, glutathione covalently binds reactive cysteine residues of reduced proteins to form protein *S*‐glutathionylation, thereby exacerbating liver fibrosis. Pirfenidone, on the other hand, has been shown to reverse protein *S*‐glutathionylation in a manner that relies on glutaredoxin‐1, thereby preventing liver fibrosis and HSC activation [131]. Additional clinical research has demonstrated that pirfenidone therapy can reduce inflammation and stiffness in patients with advanced liver fibrosis [132].

Besides the aforementioned chemical compounds, preclinical natural compounds also exhibit significant potential in inhibiting oxidative stress to improve liver fibrosis. The experimental researches revealed that quercetin, baicalin, or ginsenoside Rg1 could alleviate liver damage and fibrosis via upregulating the antioxidant factor, erythroid 2‐related factor 2 (Nrf2) [133, 134, 135, 136]. Decursin has been identified to exert hepatoprotective effects on TGF‐β‐induced experimental fibrosis by inhibiting NOX activation and Smad signaling [137]. Similarly, polydatin had recently demonstrated the reduction in oxidative stress by downregulating NOX4 enzymes and in inflammation and CD68 macrophage activation by inhibition of TLR4/NF‐κB p65 signaling pathway, which helps to suppress liver fibrosis in mouse model induced by MCD [138]. In the field of research on inhibiting oxidative stress, traditional Chinese medicine formulas also hold an important position. Xiao‐Chai‐Hu decoction activated Nrf2 to inhibit CCl_4_‐induced liver fibrosis by regulating antioxidant enzyme activity and gene expression [139].

### 5.2 Medicines Targeting Programmed Cell Death

**FIGURE 3:**
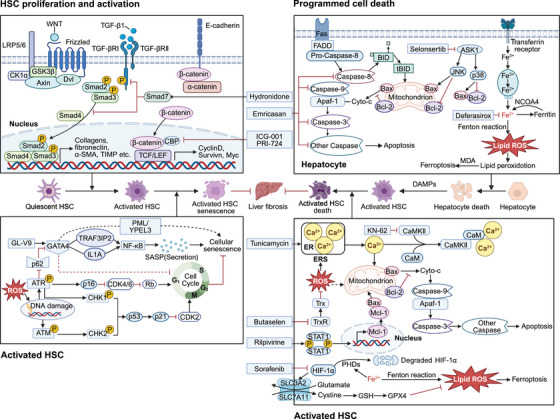
Targeting programmed cell death and preventing HSC activation or may become effective therapies for liver fibrosis. ICG‐001 and PRI‐724 inhibited the Wnt/β‐catenin pathways to halt the proliferation and activation of HSCs. Similarly, hydronidone boosted Smad 7 expression and degraded TGFβRI, preventing HSC proliferation and activation. GL‐V9 improved fibrosis and liver damage by enhancing GATA4 stability, promoting activated HSC senescence. In addition, delaying or reversing liver fibrosis can be achieved by either preventing apoptosis and ferroptosis in injured hepatic cells or enhancing these processes in activated HSCs. Emricasan, selonsertib, and deferasirox could delay or reverse liver fibrosis by preventing apoptosis and ferroptosis in injured hepatic cells. On the other hand, KN‐62, tunicamycin, butaselen, rilpivirine, and sorafenib induce apoptosis and ferroptosis in activated HSCs, assisting in the elimination of activated HSCs and preventing liver fibrosis.

Increasing evidence has demonstrated a strong correlation between the development of liver fibrosis and programmed cell death, such as apoptosis and ferroptosis. Notably, liver fibrosis can be postponed or even reversed by preventing apoptosis and ferroptosis of injured hepatic cells. Herein, we summarized the chemical compounds with potential clinical utility and traditional Chinese medicines that target programmed cell death (Figure 3). Serine/threonine kinase apoptosis signal‐regulatory kinase 1 is an essential target that initiates cell death and induces caspase activation. A Phase II trial suggested that selonsertib, a particular inhibitor of apoptosis signal‐regulatory kinase 1, could reduce LS on MRE and blood markers of necrosis and apoptosis in patients with MASH with Stage 2–3 fibrosis [140]. Nevertheless, it did not achieve the expected antifibrotic outcomes in patients with MASH‐induced fibrosis or compensated cirrhosis [141]. On the other hand, the oral pan‐caspase inhibitor emricasan (IDN‐6556) lowered total bilirubin levels and improved liver function in cirrhosis patients while also decreasing end‐stage liver disease scores [142]. However, a clinical trial showed that most patients with MASH and F1–F3 fibrosis did not experience improvement in liver histology [143]. Ferroptosis is iron‐dependent programmed cell death caused by intracellular iron overload and LPO during hepatic fibrogenesis. Deferasirox has been found to significantly improve LS and transient elastography values in β‐thalassemia major patients within 5 years through uninterrupted deferasirox chelation therapy [144].

Several traditional Chinese medicines, including Qijia‐Rougan decoction, Yin‐Chen‐Hao decoction, and Si‐Ni powder, inhibited PI3K/Akt signaling to reduce hepatocellular death in CCl_4_‐induced mice [145, 146, 147]. Moreover, the CGA formula, containing *Cordyceps sinensis mycelia* polysaccharide, gypenoside, and amygdalin, prevented CCl_4_‐induced liver fibrosis by upregulating the Bcl‐2/Bax ratio and inhibiting hepatic apoptosis [148].

### 5.3 Medicines Targeting Excessive Immune Responses

**FIGURE 4:**
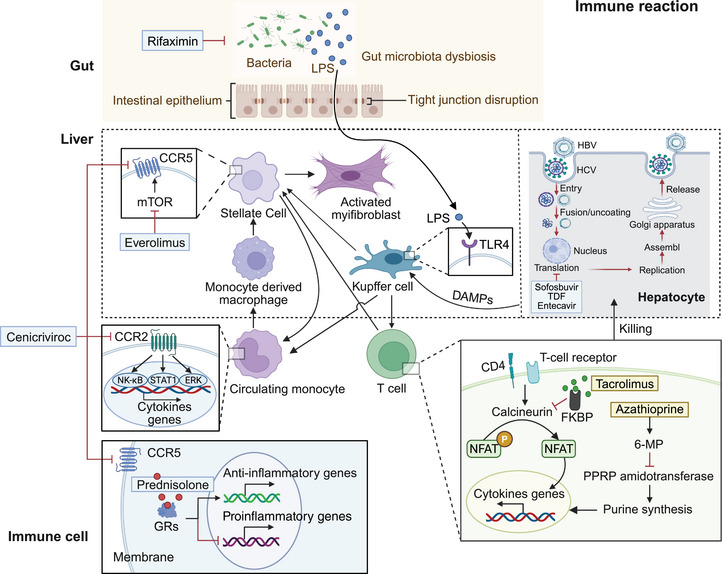
Inhibiting immune response functions as a treatment strategy for liver fibrosis. Various immune cells play a role in exacerbating liver fibrosis by influencing the inflammatory response and immune microenvironment. Cenicriviroc inhibited CCR2/5 to prevent hepatic inflammatory monocyte recruitment and alleviate liver fibrosis. Rifaximin‐α enhanced the function of the intestinal barrier, reduced inflammatory responses, and subsequently decelerated the progression of liver fibrosis. Immunosuppressants like tacrolimus, azathioprine, and short‐term prednisolone ameliorated liver fibrosis by suppressing the expression of anti‐inflammatory genes in immune cells. Everolimus reduced fibrosis progression by targeting mTOR. Additionally, sofosbuvir, TDF, and entecavir inhibited RNA polymerase, thereby dampening the immune response and improving liver fibrosis.

As we described in Section 2.2, immune cells play crucial roles in liver fibrosis. Anti‐inflammatory drugs may aid in decreasing ECM formation in liver fibrosis by restoring the immune cell composition. We summarized the chemical compounds with potential clinical utility, preclinical natural compounds and traditional Chinese medicines that achieve antifibrotic effects by inhibiting immune responses (Figure 4). A Phase II clinical trial found that individuals with MASH and fibrosis experienced improvement of more than one stage in fibrosis after undergoing treatment with cenicriviroc for 2 years [149, 150]. Moreover, in a mouse model of chemical toxicant‐induced liver fibrosis, cenicriviroc, the C–C chemokine receptors Type 2 (CCR2)/5 inhibitor, can inhibit the inflammatory monocyte recruitment and ameliorate histological fibrosis in liver by inactivating the CCR2‐signal transducer and activator of transcription 1/NF‐κB/ERK signaling pathways [151]. Within the first year of Phase III clinical trials, cenicriviroc was found to be safe and well tolerated in patients with MASH and liver fibrosis [152]. Unfortunately, it did not demonstrate the expected antifibrotic effects in a subsequent Phase III trial (NCT03028740), leading to the premature termination of the study. In addition, a Phase II study revealed that intestinal‐specific ligands, such as rifaximin‐α, a molecule of the human nuclear receptor pregnane‐X receptor, improved the function of the intestinal barrier, lowered inflammatory responses throughout the body, and then slowed the progression of alcohol‐induced liver fibrosis [153].

Natural components treat liver fibrosis by preventing oxidative stress and immune response, thereby suppressing HSC activation and proliferation. In general, TLR4 recognizes HMGB1 and induces NF‐κB transduction, ultimately leading to the assembly of NLRP3 inflammasome and increased expression of proinflammatory factors. Emerging studies discovered that several natural compounds including chlorogenic acid, astragalus polysaccharide, oxymatrine, saikosaponin d, mogroside, methoxyeugenol, oligonol and resveratrol, natural flavonoid tormentic acid, and sweroside inhibited immune response and improved liver fibrosis by inhibiting the TLR4/HMGB1/NF‐κB/NLRP3 pathway [21, 154, 155, 156, 157, 158, 159, 160, 161, 162]. Moreover, CCl_4_‐induced liver fibrosis was alleviated with the treatment of schisandrin B by downregulating NF‐κB/p38 MAPK pathway to inhibit KC polarization [163]. In addition to inflammation‐related signaling pathways, ERS is increasingly recognized to mediate inflammation and participates in the pathogenesis of liver fibrosis. Based on preclinical studies, salvianolic acid A and kaempferol demonstrated significant hepatoprotective properties against liver fibrosis by suppressing ERS [164, 165].

Traditional Chinese medicines like Huang‐Qi decoction, Danggui‐Liuhuang decoction, Qiwei‐Tiexie capsule, and Tianhuang formula improved liver fibrosis by modulating NF‐κB‐related inflammatory pathways [166, 167, 168, 169]. Moreover, the cyclic GMP–AMP synthase (cGAS)–stimulator of interferon genes (STING) pathway, a key immune signaling axis, regulates innate immunity and inflammation, playing a crucial role in gastrointestinal and liver disease progression [170, 171, 172]. Licorice extracts alleviated liver fibrosis in MASH mice by inhibiting STING oligomerization, similar to the STING inhibitor C‐176 [173]. As VENT‐03, the first cGAS–STING inhibitor, have progressed into clinical trials, the pathway demonstrated targeted potential for the clinical treatment of liver fibrosis. Recent studies revealed M1 macrophages promote fibrosis via profibrotic cytokines, while M2 macrophages reverse it. Preclinical study found Qizhu‐Yanggan and Jiawei‐Taohe‐Chengqi decoctions exerted antifibrotic effects by shifting macrophage polarization toward M2 and reducing M1 [174, 175]. Moreover, Qijia‐Rougan formula inhibits M2 polarization, while Si‐wu‐tang activates M2‐like macrophages, both ameliorating liver fibrosis [176, 177]. Interestingly, Si‐wu‐tang also mitigated MASLD via inhibiting M1 macrophage polarization [178]. These findings highlight macrophage metabolic reprogramming as a key therapeutic target for liver fibrosis. We found that compared with chemical compounds, numerous studies have indicated that natural compounds and traditional Chinese medicines exhibit advantages in inhibiting the immune response and resisting liver fibrosis.

### 5.4 Targeting the Activation of HSCs

The activation of HSCs and excessive ECM deposition are core processes in the occurrence and development of liver fibrosis. We further concluded that chemical compounds with potential clinical utility, preclinical natural compounds, and traditional Chinese medicines function in inhibiting HSC activation (Figure 3).

Chemical compounds targeting HSC activation have made significant progress in multiple clinical trials. The Wnt/β‐catenin signaling pathway, along with pathways such as TGF‐β and NF‐κB, is associated with the activation of HSCs and the transformation of HSCs into fibroblasts. A preclinical study discovered that ICG‐001, a specific Wnt/β‐catenin inhibitor, blocked HSC contraction, and the activation and deposition of collagen while suppressed macrophage infiltration and angiogenesis, which reflected the role of the Wnt/β‐catenin pathway in regulating communication among HSCs, KCs, and liver sinusoidal endothelial cell (LSECs) [179]. Another study emphasizes the effectiveness of ICG‐001 in enhancing portal angiogenesis and impeding dedifferentiation of LSECs to lessen liver fibrosis by downregulating leukocyte cell‐derived chemotaxin 2 [180]. These results indicate that Wnt/β‐catenin inhibitors are worth further development since they may help relieve liver fibrosis in various ways. What is encouraging is that, when treated with the Wnt/β‐catenin inhibitor PRI‐724, patients with cirrhosis caused by HBV and HCV showed statistically significant improvements in serum albumin levels, LS, and the model of end‐stage liver disease score [181].

During liver fibrosis, upregulated TGF‐β activates HSCs, enhances the synthesis of tissue inhibitors of MMPs, and directly promotes interstitial fibrillar collagen production. In CCl_4_‐ and 3,5‐dioxo‐1,4‐dihydrocholine‐induced animal models, hydronidone, a structurally modified version of pirfenidone designed to reduce hepatotoxicity, demonstrated an antifibrotic effect by notably increasing Smad7 expression and degrading TGFβ receptor I (TGFβRI) [182]. TGFβRI can assemble with and TGFβRII to form complexes, which in turn induce phosphorylation and nuclear translocation of Smad2/3. Phosphorylated Smad2/3 affects the transcription of target genes, ultimately leading to the activation of HSC. Simultaneously, a Phase II clinical trial revealed fibrosis regression in chronic hepatitis B patients treated with entecavir in combination with hydronidone [183]. Collectively, targeting HSCs might extrapolate to liver fibrosis caused by various liver diseases including but not limited to MASLD, PBC, HBV, and HCV.

Emerging research evidence has demonstrated that the 5‐hydroxytryptamine 2A receptor (5‐HT2AR) serves as a pivotal regulator of liver fibrosis progression. Hepatic injury induces elevated expression of 5‐HT2AR in both hepatocytes and HSCs, which in turn activates the TGF‐β/SMAD2/3 pathway, thereby exacerbating liver inflammation and promoting HSC activation. Selective 5‐HT2AR antagonists, such as sarpogrelate, have exhibited therapeutic efficacy in both in vitro and in vivo experiments, capable of reducing key fibrotic biomarkers including α‐SMA, TGF‐β, and collagen Type I [184]. In recent years, research into this target has progressively deepened this target has progressively deepened. For instance, a recent study reported that lysosome‐targeting chimeras directly regulate 5‐HT2A receptor degradation and exert antagonistic effects to ameliorate liver fibrosis [185]. More encouragingly, LY03017, a next‐generation dual‐target agent acting as both a 5‐HT2AR inverse agonist and a 5‐hydroxytryptamine 2C receptor antagonist, has obtained clearance from the US FDA to initiate clinical trials. This milestone endows the strategy of targeting 5‐HT2AR to inhibit HSC activation and ameliorate liver fibrosis with substantial clinical translational potential

In addition, several natural compounds are recently reported to protect rat liver from thioacetamide‐, CCl_4_‐, or MCD‐induced fibrosis by modulating TGF‐β/Smad signaling, including ferulic acid, gallic acid, isorhamnetin, embryonic ketone, glycyrrhizic, demethylzeylasteral, luteolin, and linderalactone [186, 187, 188, 189, 190, 191, 192]. In addition to TGF‐β, PDGF secreted during liver fibrosis encourages collagen synthesis and deposition through activation of several signaling pathways, such as ERK/MAPK pathway, upon binding to PDGFRβ on the membrane of HSCs. As expected, dihydroartemisinin, the active metabolite of artemisinin, and gomisin D against liver fibrosis induced by CCl_4_ and bile duct ligation (BDL) via the PDGF‐BB/PDGFRβ signaling pathway [193, 194]. The activation, ECM secretion, and metabolism of HSCs are also associated with the Hh pathway through the binding of Hh ligands to Patched, promoting nuclear translocation of GLI1 and the expression of Hh‐target genes. Procyanidin B2 and physalin B inhibited HSC activation and alleviated liver fibrosis through the inhibition on the Hh pathway and the activation of GLI1 [195, 196]. Tetramethylpyrazin treatment prevented HSC activation by inhibiting hepatocyte‐derived and mtDNA‐enriched extracellular vesicles (EVs) [197, 198].

The activation of HSCs and epithelial–mesenchymal transition (EMT) share a close mutual regulatory relationship in liver fibrosis, jointly promoting disease progression. Polydatin attenuates HSC proliferation and liver fibrosis by inhibiting ZEB1/miR‐203/TGF‐β/Smad signaling and suppressing sphingosine kinase 1 to attenuate EMT and HSC activation [199, 200]. Also, carnosol was observed to attenuate EMT and inhibit HSC activation through activating SIRT1/EZH2 signaling [201]. Hepatocytes undergo EMT have the ability to transform into MFs. Curcumin has been shown to inhibit EMT in hepatocytes, thereby reducing liver fibrosis in animal and in vitro models by decreasing ROS levels [202]. A clinical trial also observed the beneficial effects of curcumin supplementation in lowering serum levels of alkaline phosphatase, bilirubin, international normalized ratio, and prothrombin time, as well as disease activity scores and severity of cirrhosis in patients with cirrhosis [203]. But the recent clinical trial focused on the effect of nano‐curcumin supplementation on liver fibrosis showed that nano‐curcumin improved liver enzymes but showed no significant effect on fibrosis compared with placebo in patients with MAFLD [204].

Moreover, traditional Chinese medicine formula showed potential in targeting HSC activation. Anluo‐Huaxian pills (license number: Z20010098), a patented formula for liver fibrosis in China, improved fibrosis in CHB patients with elevated ALT and early‐stage fibrosis (≤Stage 2) within 48 h [205]. These effects were achieved by inhibiting HSC activation and proliferation. Baihe‐Wuyao, Qijia‐Rougan, Xiao‐Yao‐San, CGA formula, and compound Ku‐Shen injection blocked TGF‐β/Smad signaling, exerting antifibrotic effects in CCl_4_‐induced mice [206, 207, 208, 209, 210]. In addition, Jiawei‐Taohe‐Chengqi decoction inhibited HSC activation via the Src/ERK/Smad3 pathway [211].

### 5.5 Inducing Senescence and Programmed Death in HSC

Beyond the aforementioned inhibition of HSC activation, for already activated HSCs, alternatively, the induction of apoptosis and ferroptosis aids in their clearance and the prevention of liver fibrosis formation. Preclinical studies have demonstrated that both chemical compounds and natural compounds possess the potential to induce apoptosis and ferroptosis and ameliorate liver fibrosis. Imbalance of calcium ion (Ca^2+^) levels in HSCs can trigger ERS and facilitate cell apoptosis. Findings from preclinical trials demonstrated that the Ca^2+^ kinase II inhibitor KN‐62 and the ERS inducer tunicamycin both elevated intracellular Ca^2+^ levels, leading to increased expression of ERS protein GRP78 and apoptotic protein and further ultimately inhibited HSC proliferation and promoted apoptosis of activated HSCs [212, 213]. Furthermore, butaselen therapy has been shown to potentially prevent chronic liver inflammation, fibrosis and cirrhosis in murine models. This effect is believed to be associated with the inhibition of thioredoxin reductase activity, leading to elevated levels of ROS, G2/M cell cycle arrest, and induction of apoptosis [214]. Natural compounds can also induce HSC apoptosis to alleviate liver fibrosis. Saikosaponin A and oroxylin A triggered mitochondrial dysfunction and HSC apoptosis involving caspase‐3‐dependent and independent pathways modulating the Bcl‐2 family [215]. Oroxylin A induced apoptosis of activated HSCs via ERS, blocked the glycolysis, hence alleviating liver fibrosis [216]. Interestingly, rilpivirine, a popular antihuman immunodeficiency virus medication, was found to specifically induce activated HSC death in a STAT1‐dependent manner, improving liver fibrosis in a rat model of nonviral‐caused liver fibrosis.

Another understanding of how certain chemical/natural compounds combat liver fibrosis involves their regulation of ferroptosis. Sorafenib caused ferroptosis events in HSCs by regulating the hypoxia‐inducible factor‐1α/solute carrier family 7 member 11 pathway, ultimately reducing fibrosis and liver damage of CCl_4_‐induced mouse models [217]. Natural compounds including wogonoside, artemether, schisandrin B, and isoliquiritigenin exerted antifibrosis efficacy in vivo and in vitro by inducing HSC ferroptosis [218, 219, 220, 221, 222]. An ellagic acid was reported to inhibit oxidative stress and the apoptotic pathway to ameliorate iron‐overload‐induced hepatotoxicity as well as block the formation of soluble NSF attachment protein receptor complex formation to trigger ferroportin‐dependent ferroptosis in vivo [223, 224].

Due to the potential reactivation of drug‐induced inactivated HSCs, strategies aimed at inhibiting HSC activation have shown limited success. Recent research suggests that inducing HSC senescence has emerged as another therapeutic strategy targeting HSCs [225]. This approach significantly improves serum biochemical markers in chemical‐induced and surgical‐induced liver fibrosis mouse models, reduces the proportion of activated HSCs, and increases the proportion of quiescent HSCs. A newly synthesized flavonoid derivative GL‐V9 was confirmed to improve fibrosis and liver damage in mouse models induced by BDL and CCl_4_ via enhancing GATA4 stability, thereby promoting HSC senescence, preventing HSC proliferation and activation [226]. Moreover, the Hippo pathway modulates the progression of liver fibrosis by regulating cellular proliferation and the synthesis of ECM components, which has also been reported to be highly related to HSC senescence. YAP knockout specifically in HSCs induces cellular senescence and protects damaged liver tissue, alleviating fibrosis [227]. The small molecular targeting YAP activity in HSC may benefit the development of liver fibrosis, which are worthy for further investigation.

Several natural compounds also showed significance effects in inducing HSC senescence. Oroxylin A activates ferritinophagy to induce HSC senescence against liver fibrosis by regulating cGAS DNA hypermethylation induced by methionine metabolism [228, 229]. Moreover, due to the unique cytoskeletal homeostasis in HSCs, ligustilide disrupts cytoskeletal remodeling to compromise nuclear integrity with YAP participation, and further activates the cGAS–STING pathway, thereby specifically targeting HSC senescence and ultimately alleviating liver fibrosis [225].

## 6 Emerging and Unconventional Therapies

The gut–liver axis theory has driven progress in the diagnosis, prognosis, and treatment of liver fibrosis. Intestinal products can be directly transported to the liver via the bile duct, portal vein, and systemic circulation, while the liver in turn exerts regulatory effects on the intestine, forming a mutually interactive relationship between the two organs. The adult gut microbiota harbors approximately 10^13^ to 10^14^ microorganisms, and their translocation into the bloodstream via the gut–liver axis can aggravate liver fibrosis. Liver fibrosis is strongly modulated by various intestinal factors, including intestinal bacteria and their metabolites. Moreover, both intestinal dysbiosis and intestinal barrier dysfunction exacerbate liver fibrosis through the aberrant regulation of the gut–liver axis.

Intestinal bacterial imbalance disturbs the gut–liver axis to drive liver fibrosis. Several reviews have summarized the alterations in the gut microbiota of patients with liver fibrosis and pointed out that oral probiotics exhibit significant efficacy in treating liver fibrosis [230, 231]. Mechanistically, probiotics alleviate liver fibrosis by modulating bile acid metabolism, inhibiting oxidative stress, and suppressing inflammation. For instance, oral administration of the probiotic *Lactobacillus rhamnosus GG* prevents liver fibrosis by upregulating the intestinal FXR/FGF15 axis, inhibiting hepatic bile acid synthesis, and promoting bile acid excretion [232]. Moreover, oral administration of *Akkermansia muciniphila* reduces gut‐derived inflammatory responses, thereby alleviating liver injury and inflammation [233]. Notably, several reviews consistently support that a reduction in butyrate‐producing bacteria is a common ecological feature in liver fibrosis [234, 235]. Butyrate inhibits the inflammation‐fibroblast axis through barrier function regulation and immunometabolism pathways. For example, the butyrate‐producing bacterium *Parabacteroides distasonis* improves liver fibrosis by remodeling intestinal bile acid metabolism and inhibiting hepatocyte pyroptosis [236]. Therefore, reconstructing the butyrate‐producing intestinal ecosystem may serve as a promising research strategy for effective antifibrotic therapy in the future.

Studies have demonstrated that the application of probiotic combinations for the treatment of liver fibrosis can significantly slow down disease progression and enhance the proliferation of beneficial intestinal bacteria. Consequently, the antifibrotic effects of probiotic products have been further investigated in depth. Administration of Mutaflor, a probiotic product composed of multiple probiotic strains, minimizes the biochemical and histopathological changes induced by MASLD/MASH [237]. MCP BCMC Strains, a probiotic product containing six live strains of *Lactobacillus* and *Bifidobacterium*, could stabilize mucosal immune function, protect MASLD patients from increased intestinal permeability, and exert a complementary therapeutic effect on MASLD [238]. However, probiotic products still face many practical limitations in the clinical treatment of liver fibrosis. First, strain specificity and individual variation are notable issues. The antifibrotic effects of probiotics rely heavily on the specific strain used, and patients with liver fibrosis differ greatly in baseline gut microbiota composition, etiology, and genetic background. As a result, the same product may exhibit different therapeutic effects in different patients. Second, intestinal colonization efficiency is limit in patients with liver fibrosis. Moreover, these patients often present with impaired intestinal barrier function and dysregulated intestinal motility, which make it difficult for exogenous probiotics to stably colonize the gut. As a result, long‐term continuous administration is needed to maintain only mild therapeutic effects. Additionally, there is a lack of standardized treatment criteria for the optimal dosage, treatment duration, and administration route of probiotics, which further limits their clinical application.

Additionally, cell‐based therapies, including stem cell or macrophages therapy, have presented both potential and risks in antifibrotic treatments, supported by a growing body of evidence from both experimental and clinical studies. As reported, both mesenchymal stromal cells (MSCs)‐ and MSC‐derived EVs therapy for patients significantly improved liver function, promoted the repair of injured liver tissue, reduced proinflammatory factors, and increased anti‐inflammatory cytokines compared with the placebo control group [239]. Although the reinfusion of CD133^+^ stem/progenitor cells in patients with end‐stage liver disease is feasible and safe, the use of G‐CSF, with or without CD133^+^ stem‐cell infusion, did not improve liver dysfunction or fibrosis in patients [240, 241]. Furthermore, it may be associated with a higher frequency of adverse events compared with standard care. Moreover, autologous macrophage therapy for liver cirrhosis is also in the stage of clinical trials [242]. Thus, cell‐based therapies have been considered as an alternative treatment.

## 7 Challenges in the Development of Antifibrotic Drug

**FIGURE 5:**
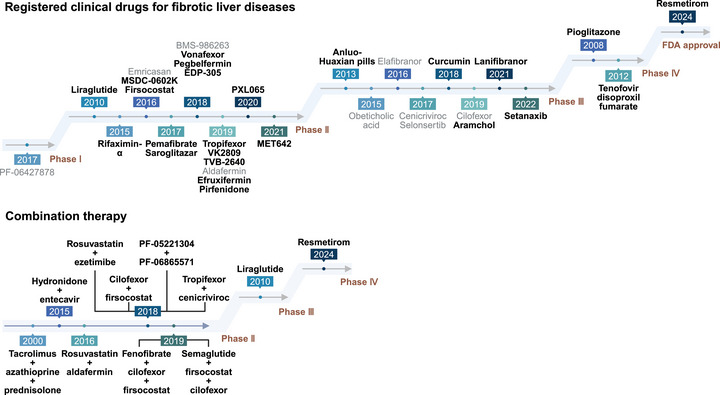
The current status of registered clinical drugs for fibrotic liver diseases. Drugs highlighted in gray indicate that their clinical trials for the treatment of fibrotic disease have been abandoned due to the failure to achieve expected results or other reasons.

Certain limitations still hinder the development of chemical compounds, natural compounds, and traditional Chinese medicinal formulas. Currently, the clinical treatment of liver fibrosis primarily focuses on eliminating pathogenic factors such as viruses, ethanol, and metabolic dysfunction. In addition, clinical studies have found that targeting the activation of HSCs and the accumulation of ECM can improve or even reverse liver fibrosis. As the mechanisms of liver fibrosis are further elucidated, many novel targets, such as FGF21 and DGAT2, have been gradually discovered, and their corresponding inhibitors have entered clinical trials. However, several clinical trials developed for fibrotic liver diseases have been terminated due to their failure to achieve the primary treatment endpoints (Figure 5 and Table 1).

**TABLE 1:** The current status of registered clinical drugs for fibrotic liver diseases.

Medication	Target/mechanism	State date	Current status	Condition	Adverse events	Latest results	References
Monotherapy							
Resmetirom	THR‐β agonist	2024	US FDA approval	MASH	Diarrhea and nausea	Reduction in MASLD activity score (≥2 points) or fibrosis score (≥1 points)	—
VK2809	THR‐β agonist	2019	Phase II (NCT04173065); active, not recruiting	MASH and fibrosis (F1–F3)	No serious adverse events	—	—
Pemafibrate	PPAR‐α agonist	2017	Phase II (NCT03350165); completed	MASLD	Diarrhea, back pain	Reduction in liver stiffness and levels of ALT and LDL‐C but not liver fat content	[42]
Pioglitazone	PPAR‐α agonist	2008	Phase IV (NCT00994682); completed	MASLD, MASLD, and T2DM	Edema and weight gain	Reduction in MASLD score (≥2 points) and fibrosis score	[243]
PXL065	PPAR‐γ agonist	2020	Phase II (NCT04321343); completed	MASH	Headache, nausea, and abdominal pain	Reduction in liver fat content and fibrosis scores	[44]
Saroglitazar	PPAR‐α/γ agonist	2017	Phase II (NCT03061721); completed	MASLD/MASH	Diarrhea, cough, abdominal pain, rash, and bronchitis	Improvement in ALT level, liver fat content, insulin resistance, and atherogenic dyslipidemia	[49]
Elafibranor	PPAR‐α/δ agonist	2016	Phase IIb (NCT01694849); completed	MASH	Increase in creatinine levels	Improvement in liver fibrosis without worsening of MASH	[50]
Lanifibranor (IVA337)	PPAR‐α/γ/δ agonist	2021	Phase III (NCT04849728); recruiting	MASH and fibrosis (F2–F3)	Nausea, edema, and headache	—	—
MSDC‐0602K	MPC inhibitor and PPAR‐γ agonist	2016	Phase II (NCT02784444); completed	MASH and fibrosis (F1–F3)	—	Improvement in glucose metabolism and liver enzyme	[45]
Deferasirox	Iron chelator	2008	Phase ΙV (NCT01335035); completed	Beta‐thalassemia major	Diarrhea, constipation, and rash	Reversing or stabilizing liver fibrosis progression	[144]
Tenofovir disoproxil fumarate	RNA polymerase inhibitor	2012	Phase IV (NCT01522625); completed	CHB	Renal function deterioration	Improvement in liver fibrosis	[120]
Anluo‐Huaxian pills	—	2013	Phase III (ChiCTR‐IOR‐14005474); completed	Chronic hepatitis B and liver fibrosis	Nausea, diarrhea, and pruritus	Improvement in liver fibrosis	[205]
OCA	FXR agonist	2015	Phase III (NCT02548351); terminated	Liver fibrosis (F1–F3)	Pruritus	Achievement of the fibrosis improvement endpoint but not MASH resolution endpoint	[98]
EDP‐305	FXR agonist	2018	Phase II (NCT03421431); completed	MASH fibrosis without cirrhosis	Pruritus	Reduction in ALT level and liver fat content	[101]
Cilofexor	FXR agonist	2019	Phase III (NCT03890120); terminated	PSC	Nausea, headache, and fatigue	Not significant improvement in fibrosis	[104]
Vonafexor (EYP001)	FXR agonist	2018	Phase II (NCT03812029); completed	Liver fibrosis (F2–F3)	Pruritus	Improvement in liver fat, body weight, and liver enzymes	[107]
MET642	FXR agonist	2021	Phase II (NCT04773964); completed	MASH	High levels of ALP	—	—
Selonsertib	ASK1 inhibitor	2017	Phase III (NCT03053050 and NCT03053063); terminated	MASH and fibrosis (F3–F4)	Diarrhea, nasopharyngitis, and nausea	Not significant improvement in fibrosis	[140]
Cenicriviroc	CCR2/5 inhibitor	2017	Phase III (NCT03028740); terminated	MASH and fibrosis (F2–F3)	Nausea, sinusitis, and nephrolithiasis	Not significant improvement in fibrosis	[152]
Curcumin	—	2018	Phase III (IRCT20180802040678N1); completed	Liver cirrhosis	Nausea, rash, and anticoagulation	Reduction in liver cirrhosis disease activity scores	[202]
Aramchol	SCD1 inhibitor	2019	Phase III (NCT04104321); suspended	MASH and fibrosis (F2–F3)	Nausea, diarrhea, and myalgia	Improvement in NASH without worsening of fibrosis	—
Setanaxib	NOX1/4 inhibitor	2022	Phase III (NCT05014672); active, not recruiting	PBC or liver stiffness	Pruritus, nausea, and fatigue	—	—
Liraglutide	GLP‐1R agonist	2010	Phase II (NCT01237119); completed	MASH	Nausea, hypoglycemia	Improvement of MASH without fibrosis worsening	[77]
Rifaximin‐α	PXR activator	2015	Phase II (EudraCT 2014–001856‐51); completed	Alcohol‐related liver disease	Pruritus, nausea	Improvement in liver fibrosis	[153]
Emricasan (IDN‐6556)	Pan‐caspase inhibitor	2016	Phase II (NCT02686762); completed	NASH and fibrosis (F1–F3)	Diarrhea, sinusitis, and back pain	Exacerbation of hepatic fibrosis and ballooning	[142]
BMS‐986263	Silencing HSP47 mRNA	2018	Phase II (NCT03420768); completed	Advanced liver fibrosis	Infusion‐related reactions	Positive but limited improvements in the METAVIR and Ishak scores	[29]
TVB‐2640	FASN inhibitor	2019	Phase II (NCT03938246); completed	MASH	Headache, peripheral edema, rash, upper respiratory tract infection, bronchitis, diarrhea, nausea, urinary tract infection, and hyper‐triglyceridemia	Reduction of liver fat and improvement of fibrotic markers	[64]
Pegbelfermin	FGF21 analog	2018	Phase II (NCT03486899); completed	MASH and fibrosis (F3)	Nausea and diarrhea	Reduction in fibrosis score (≥1 points) and MASLD score	[53]
Efruxifermin	FGF21 analog	2019	Phase II (NCT03976401); completed	MASH	Gastrointestinal	Reduction in hepatic fat fraction	[54]
Aldafermin (NGM283)	FGF21 analog	2019	Phase II (NCT03912532); completed	MASH and liver fibrosis (F2–F3)	Diarrhea	Not significant improvement in fibrosis stage (≥1)	[116]
NGM282	FGF19 analog	2016	Phase II (NCT02443116); completed	MASH	Headache, nausea, vomiting, diarrhea, injection site reactions, and myalgia	Improvement of NGM282‐associated cholesterol elevation	[115]
Pirfenidone	TGF‐β inhibitor	2019	Phase II (NCT04099407); completed	Liver fibrosis or chronic liver disease	Nausea, dyspepsia, diarrhea, rash	Improvement in liver fibrosis and the levels of ALT, AST, and TGF‐β	[132]
PF‐06427878	DGAT2 inhibitor	2017	Phase I (NCT02855177 and NCT02391623); completed	Healthy adult	Diarrhea, abdominal pain, headache, dyspepsia, nasal dryness, constipation, flatulence, gastroenteritis	Improvement markers of liver function and reduction of hepatic steatosis	[73]
Combination therapy							
Entecavir + Biejia‐Ruangan compound	RNA polymerase inhibitor + traditional Chinese medicine	2013	Phase IV (NCT01965418); unknown status	Liver fibrosis and CHB	Headache, diarrhea, and nausea	Slowed progression of severe fibrosis	[118]
Hydronidone + entecavir	TGF‐β inhibitor + RNA polymerase inhibitor	2015	Phase II (NCT02499562); completed	Liver fibrosis	Dizzy, rash, diseases of the gastrointestinal system, hypertension	Improvement in histological features of liver fibrosis	[183]
Exenatide + dapagliflozin	GLP‐1 analogue + SGLT2 inhibitor	2014	Phase III (NCT02229396); completed	Type 2 diabetes mellitus	Acute renal failure, gastrointestinal disorder	Improvement in hepatic steatosis and fibrosis markers	[86]
Rosuvastatin + ezetimibe	HMG‐CoA reductase inhibitor + NPC1L1 inhibitor	2018	Phase II (NCT03434613); completed	MASLD	No significant adverse event	Reduction of liver fat but not liver fibrosis	[69]
Fenofibrate + cilofexor + firsocostat	PPARα agonist + ACC inhibition + FXR agonist	2019	Phase II (NCT02781584); completed	MASH with elevated triglycerides	Anemia and appendicitis	Improvement in triglyceride elevations induced by cilofexor/firsocostat	[244]
Semaglutide + firsocostat + cilofexor	GLP‐1 analogue + ACC inhibitor + FXR agonist	2019	Phase II (NCT03987074); completed	MASH (F2–F3)	Gastrointestinal, pruritus	Improvement in liver steatosis and biochemistry	[82]
Tropifexor + cenicriviroc	FXR agonist + CCR2/5 inhibitor	2018	Phase II (NCT03517540); completed	MASH	Pruritus, fatigue, nasopharyngitis	Improvement in ALT level, body weight, and fibrosis stage/steatohepatitis	[106]
Cilofexor + firsocostat	ACC inhibitor + FXR agonist	2018	Phase II (NCT03449446); completed	Liver fibrosis (F3–F4)	Pruritus	Improvement in fibrosis stage (≥1)	[67]
PF‐05221304 + PF‐06865571	ACC inhibitor + DGAT2 inhibitor	2018	Phase II (NCT03776175); completed	MASH	High levels of triglycerides	Reduction in liver fat and PF‐05221304‐induced triglycerides elevation	[70]
Tacrolimus + azathioprine + prednisolone	Calcineurin inhibitor	2000	Phase II (ISRCTN94834276); completed	HCV cirrhosis receiving cadaveric grafts	Ascites, bleeding, varices, encephalopathy	Slowed progression of severe fibrosis	[122]

For example, emricasan, BMS‐986263, and aldafermin were discontinued during Phase II clinical trials for MASH and liver fibrosis as they did not meet the primary endpoints. Similarly, elafibranor, cenicriviroc, selonsertib, and cilofexor were stopped in phase III trials for MASH or PBC patients with liver fibrosis due to the lack of expected outcomes. This may be due to the fact that the pathogenesis of MASH involves multifactorial pathological mechanisms, and inhibition of a single target (for example, ASK1) is difficult to fully block the progression of the disease. Moreover, the failure of cenicriviroc may be attributable to patient selection bias, namely, the exclusion of cirrhotic patients, who are precisely the population most in need of antifibrotic therapy. Unlike above, the Phase III trial of OCA in MASH and liver fibrosis patients was halted primarily because of severe adverse reactions, such as pruritus. Hence, it is widely recognized that single‐drug therapies may not be adequate for effectively managing liver disorders in clinical practice.

Moreover, the bioavailability of chemical compounds and natural compounds imposes constraints on their applications. During the aggravation of liver fibrosis, accumulated ECM and closure of endothelial fenestrae constitute a major and almost impenetrable physical barrier inhibit the delivery of antifibrotic agents to the liver [245]. Delivery of antifibrotic agents to the fibrotic environments is also reduced by the reduced space of Disse. On the other hand, the bioavailability of some small‐molecule drugs also hinders their clinical application. For instance, silymarin and berberine exhibit antifibrotic effects in liver but have extremely low oral bioavailability, which constitutes a key limitation leading to weak or inconsistent efficacy during clinical translation [246, 247].

For traditional Chinese medicine, the lack of standardized preparation methods and standardized evaluation for their clinical efficacy have significantly limit their applications. Fortunately, this situation is gradually improving. Clinical studies have shown that nearly 10 traditional Chinese medicines exhibit significant antifibrotic effects. For instance, high‐level evidence‐based medicine evidence supports that traditional Chinese medicines such as Biejia‐Ruangan compound, Fuzheng Huayu formula, and AnluoHuaxian pill, possess the ability to improve or even reverse liver fibrosis [248]. With the continuous accumulation of findings from clinical studies meeting international standards, the application of traditional Chinese medicine in antifibrotic therapy will transition from an experience‐driven approach to evidence‐based clinical practice.

## 8 Conclusion and Future Perspectives

### 8.1 The Plausible Application of Precision Medicine

Given disease heterogeneity, precision medicine seeks the most effective, side‐effect‐free therapy via individual variability in genes, environment, and lifestyle. In this framework, clinical features, genetics, and omics advance molecular disease understanding to enable effective treatments. Additionally, novel technologies enabling specific organ/gene targeting are also a form of precision medicine. In the context of MASLD, precision medicine adopts the 4P care model, encompassing preventive, predictive, personalized, and participatory approaches [249]. This model takes into account the distinctive interplay of each patient's disease manifestations, genetic makeup, microbiota composition, and lifestyle factors. Moreover, diverse technological advances facilitate the development precision medicine, with organoids and omics emerging as core integrated tools in precision and translational medicine [250]. Omics technologies such as single‐cell and spatial transcriptomics contribute to deeply underlying mechanisms of liver fibrosis. Also, emerging 2D and 3D in vitro models, together with precision‐cut liver slices, enable the personalized in vitro simulation of liver fibrosis characteristics in different patients.

Specifically, nucleotide‐based therapeutics, namely, small interfering RNA and antisense oligonucleotides, are developing rapidly in the field of liver fibrosis and have been summarized in detail in review [251]. Gene mutations and polymorphisms are closely associated with increased susceptibility to liver injury development and elevated risk of HCC, thereby rendering these genes as pivotal therapeutic targets for disease management. The modified oligonucleotides can specifically and effectively mediate posttranscriptional hepatic gene silencing for clinical purposes, demonstrating properties of high efficacy, low toxicity, high specificity, and high tissue distribution. Currently, several oligonucleotide‐based drugs targeting *PNPLA3* are undergoing early‐phase clinical trials for the treatment of MASLD.

### 8.2 The Development of Combination Therapies

Combination therapies are anticipated to surpass monotherapies and become the main focus of future therapeutic advancements. Drug combination therapies in clinical trials have been summarized in Table 1. Moreover, the combinations of ezetimibe and rosuvastatin, or semaglutide, cilofexor, and firsocostat, have respectively shown greater benefits in patients with MASH and liver fibrosis compared with monotherapies. In addition to better efficacy, fenofibrate helped reduce cilofexor/firsocostat‐induced elevated TG levels when combined with cilofexor and firsocostat, while rosuvastatin mitigated lipid changes associated with NGM282 in MASH patients. It is worth noting that the Biejia‐Ruangan compound, the first orally prescribed antifibrotic traditional Chinese medicine approved by the China FDA, has shown better effect in halting fibrotic progression when used in combination with entecavir for patients with CHB and advanced fibrosis or cirrhosis, compared with entecavir alone. This case has inspired global scientists to further investigate certain traditional Chinese medicines that have been widely used clinically, such as Anluo‐Huaxian pills, through evidence‐based research or combination therapy design.

In summary, the combination modes can be categorized into three types: (1) the combination of etiological treatment and antifibrotic mechanism‐based treatment, which serves as the core combination mode; (2) combinations where the coadministered drugs target the same etiology but act on different targets, enhancing etiological therapeutic efficacy through synergistic effects; (3) combinations where the coadministered drugs do not directly target a definite etiology but instead act on distinct key mechanisms of fibrosis progression, strengthening antifibrotic effects via target complementarity.

### 8.3 The Discovery of Novel Druggable Target

Single‐cell RNA sequencing technology can precisely analyze gene expression and heterogeneity across different cell types, revealing the dynamic changes in cell subpopulations and the expression of related genes during liver fibrosis. This, in turn, helps to understand the functions, interactions, and contributions of various cell subtypes to disease progression in liver fibrosis. A comprehensive understanding of the mechanisms driving liver fibrosis progression is essential for the advancement of novel therapeutic strategies.

While significant research has been conducted on the roles of hepatocytes, immune cells, and HSCs in liver fibrosis, the impact of LSECs on this process remains inadequately explored. As the guardians of hepatic homeostasis, LSECs have the potential to modulate immune responses following acute and chronic liver injury, thereby preserving liver function. However, several studies have focused on the functions of LSEC in liver fibrosis and indicate that LSECs experience dedifferentiation, known as LSEC capillarization, in the early stages of liver fibrosis even prior to KC activation [252, 253, 254]. This not only diminishes their protective properties, impairing their ability to produce NO, which inhibits HSC activation, but also contributes to hepatic angiogenesis, inflammation, and fibrosis. Although substances released from liver adipose tissue and the intestines play a role in mediating LSEC capillarization, the mechanisms that induce LSEC capillarization during liver fibrosis are currently not fully understood. In recent years, the role of microvascular thrombosis has been postulated as one of the main mechanisms promoting liver fibrosis. A direct role for microvascular thrombosis in promoting LSEC dysfunction in liver fibrosis has been extensively discussed in review [255]. Due to the detrimental impacts induced by microvascular thrombosis, dedifferentiated LSECs no longer express molecules that inhibit platelet activation, coagulation, and thrombosis, but instead exhibit altered expression of pro‐ and antithrombotic factors. Furthermore, the expression of thrombomodulin, NO, and prostaglandin I2 is attenuated in dysfunctional LSECs, which additionally expose Von Willebrand factor, integrins, and other receptors that interact with activated platelets to induce clot formation.

With the exception of LSEC capillarization, the reduction or loss of LSEC fenestrae is a significant factor that contributes to the progression of liver fibrosis. This is due to the decreased efficiency of blood filtration in the hepatic sinusoids, which impairs the liver's ability to eliminate harmful substances. Research aimed at restoring the reduction of LSEC fenestrae and improving material exchange efficiency in liver tissue shows promise for enhancing liver fibrosis treatments. For instance, Xuefu‐Zhuyu decoction, Dahuang‐Zhechong pills and Si‐wu‐tang demonstrated antifibrotic properties by protecting LSEC function to inhibit angiogenesis in the fibrotic liver [256, 257, 258]. However, a current research gap exists in fully understanding the mechanism of fenestration in LSECs, which hampers the development of effective treatment approaches. Additionally, it is unclear whether the phenotype and function of LSECs vary among different causes of liver fibrosis. Beyond examining liver cell types, it is crucial to consider how dysfunction in other organs impacts liver fibrosis. This underscores the importance of investigating the communication between organs, diverse cell types, and shared signaling pathways in future studies.

Target the autophagy of various cells against liver fibrosis is another novel strategy. First, as concluded in a review [259], inducing HSC autophagy exerts antifibrotic effects through multiple mechanisms, including stimulating ferroptosis, apoptosis, or senescence, reducing Type I collagen accumulation, inhibiting the release of EVs, and degrading profibrotic factors. In addition, recent studies have demonstrated that during liver injury, macrophage autophagy can inhibit neutrophil accumulation and monocyte infiltration, while reducing hepatocyte apoptosis and inflammatory responses, thus protecting hepatic tissue and attenuating liver fibrosis [260]. In addition, inhibition of hepatocyte autophagy increases TNF‐dependent liver injury by inducing hepatocyte apoptosis [261]. Future studies focusing on clarifying the regulatory mechanisms of autophagy and exploring targeted modulators with high specificity will provide new breakthroughs for clinical antifibrotic treatment.

### 8.4 The Development of Advanced Drug Delivery System

Drug delivery systems, especially those utilizing nanomaterials, show promise for innovative therapeutic strategies in liver disease treatment. These systems can improve drug effectiveness and reduce side effects by modifying drug delivery and release sites, changing drug metabolism, enhancing sustained release properties, controlling drug release, and overcoming physiological barriers. The range of nanoparticle systems includes inorganic nanoparticles, liposomes, and nano‐micelles. Inorganic nanoparticles may have cytotoxic effects, which can be minimized by incorporating biocompatible components. Liposomes and micelles, considered first‐generation nanoparticles, are more commonly used due to their lower toxicity. Second‐generation nanomedicines like solid lipid nanoparticles and nanostructured lipid carriers are being developed for liver fibrosis treatment. Their stable structures promote extended drug release and decrease unwanted cellular uptake, optimizing nanoparticle performance. Peng et al. have summarized the research progress of nano‐delivery vectors targeting cells related to the process of liver fibrosis [262].

In the past few years, nanodelivery strategies targeting HSCs has remained the core focus of anti‐liver fibrosis research. Thus, we updated the newest researches. Some nanomedicines target HSCs to release antifibrotic drugs and then exert the antifibrotic effect through the inhibition on the expression of specific genes, peptides, and proteins involved in HSC proliferation and activation, including heat shock protein (HSP) 47 mRNA, IL‐11, and TGF‐β. In a Phase II trial, using lipid nanoparticles to deliver small interfering RNA that targets HSP47 mRNA, BMS‐986263 treatment demonstrated positive but limited improvements in the METAVIR and Ishak scores in patients with HCV‐sustained virologic response and advanced liver fibrosis within 36 weeks [29]. In addition, calcipotriol, a synthetic low‐calcemic vitamin D receptor agonist, has shown potential in neutralizing TGF‐β and reducing liver fibrosis. However, its clinical application for this purpose is hindered by suboptimal pharmacokinetics, biodistribution challenges, and the risk of side effects like hypercalcemia. A recent study developed a self‐assembled drug nanoparticle incorporating calcipotriol for systemic administration, successfully aiding in the regression of liver fibrosis without causing hypercalcemia [263]. Similarly, galunisertib (GLY), as a TGF‐β receptor Type I kinase inhibitor, has antifibrotic properties and showed therapeutic effects in patients with locally advanced rectal cancer in a clinical trial [264]. However, GLY was ineffective in reducing the stage of liver fibrosis, which might have resulted from blood dilution, retention by the immune system or loss in the gastrointestinal tract. The encapsulation of GLY in polymeric polygalacturonic–polyacrylic acid nanomicelles can improve the bioavailability and show better effects on reducing collagen deposition and HSC activation [265]. Silibinin, a flavonoid lignan extracted from the seeds of the herb milk thistle *Silybum marianum (L.) Gaertn (Carduus marianus L., Asteraceae)* can reverse HSC activation and proliferation. Researchers designed silibinin albumin nanocrystals by encapsulating silibinin within albumin‐coated nanocrystals. This nanomedication was found to shield silibinin from clearance by the mononuclear phagocytic system and specifically target activated HSCs, significantly improving the bioavailability and effectiveness of silibinin [266]. In addition to using silibinin alone, researchers have devised a novel strategy involving the coencapsulation of two antifibrosis medications, silibinin and sorafenib, within silica cross‐linked micelles that have been modified with the peptide CTCE9908. This nanosystem is capable of specifically targeting the overexpressed receptor C–X–C chemokine receptor Type 4 in activated HSCs, thereby enhancing the alleviation of liver fibrosis symptoms [267]. Similarly, a separate study investigated the coencapsulation of collagenase Type I and silibinin in nanoparticles coated with chondroitin sulfate. Collagenase Type I was observed to break down excess collagen and disrupt the ECM collagen barrier, thereby enhancing silibinin penetration into ECM accumulation tissues and improving therapeutic efficacy [268].

Due to the potential of collagenase Type I in aiding nanodelivery systems in overcoming the ECM barrier in liver fibrosis environment, it has become a crucial candidate for combination with other therapeutic agents within nanodelivery systems. For example, collagenase Type I and probucol‐loaded nanoparticles, collagenase Type I and retinol codecorated polymeric micelle loaded with nilotinib, and codelivery with sorafenib using glycyrrhetinic acid‐conjugated prodrug with collagenase Type I decoration were developed to enhance ECM degradation and target HSCs in liver fibrosis therapy [269, 270, 271]. In addition to collagenase Type I, certain nanomedicines have been discovered to effectively reduce the expression of Type I collagen, leading to an improvement in liver fibrosis. One innovative approach involved the development of Golgi apparatus‐targeted chondroitin‐modified nanomicelle, which encapsulated retinoic acid and doxorubicin into chondroitin sulfate‐deoxycholic acid conjugate. This nanomicelle was found to accumulate in the Golgi apparatus of activated HSCs, disrupting its structure and leading to decreased Type I collagen production in a rat model of primary liver fibrosis [272]. Similar to chondroitin sulfate, hyaluronic acid can specifically interact with CD44, which is overexpressed on activated HSCs. In light of this, researchers have formulated hyaluronic acid–bilirubin nanoparticles, consisting of endogenous bilirubin with antioxidant and anti‐inflammatory properties and hyaluronic acid, to inhibit collagen production [273]. Additionally, a nanoparticle system containing antisense oligonucleotide‐laden retinol‐conjugated polyetherimine effectively recruited retinol binding protein 4 in its corona components, enabling direct delivery to HSCs and decreased the expression of Type I collagen [274].

Despite significant advancements in nanomedicine, only a limited number of nanoparticles have undergone clinical trials for liver fibrosis, with the main translational challenge lying in the lack of substantial efficacy improvements. Beyond efficacy issues, nanomedicine also faces hurdles including complex formulation processes and inherent instability, which lead to significant batch‐to‐batch variability and hinder production standardization and clinical application. Additionally, it may interfere with immune responses and cause potential side effects, while precise delivery to specific cell types and minimization of off‐target effects remain unmet needs. Further research is urgently required to address these barriers.

### 8.5 Conclusion

In summary, liver fibrosis remains an unmet clinical need on a global scale. This review provides a comprehensive overview of the etiologies, diagnostic approaches, pathogenic mechanisms of liver fibrosis. It also focuses on recent advances in liver fibrosis treatment, with an emphasis on the efficacy and mechanisms of chemical molecules in clinical trials. Furthermore, this review highlights the significance of exploring novel pathogenic targets, the directions of emerging and future therapeutic strategies, and the challenges in the development of antifibrotic drugs.

## Author Contributions

**Xiaojiaoyang Li**: conceptualization, funding acquisition, project administration, supervision, writing – review and editing, and resources. **Runping Liu**: conceptualization, funding acquisition, supervision, writing – original draft, and writing – review and editing. **Jiaorong Qu**: data curation, funding acquisition, investigation, writing – original draft, and writing – review and editing. **Wenqing Qin**: formal analysis, investigation, and visualization. **Minghang Dong**: data curation, formal analysis, and investigation. **Zhi Ma**: data curation, investigation, validation, and writing – original draft. **Si Li**: data curation, investigation, visualization, and writing – original draft. **Ranyun Chen**: investigation and visualization. **Changmeng Li**: investigation and visualization. All authors have read and approved the final manuscript.

## Funding

This work was supported by National Natural Science Foundation of China (Grant NO. 82322075 to RL, NO. 82404984 to JQ, Grant NO. 82274186 to XL); Young Talents Promotion Project of China Association of Traditional Chinese Medicine (No. 2025‐QNRC2‐A04 to JQ); High‐level traditional Chinese medicine key subjects construction project of National Administration of Traditional Chinese Medicine‐Beijing University of Chinese Medicine, Chinese Medicine Epidemic Disease (Grant NO. zyyzdxk‐2023264 to XL).

## Ethics Statement

The authors have nothing to report.

## Conflicts of Interest

The authors declare no conflicts of interest.

## Data Availability

The authors have nothing to report.
